# Automated analysis of genomic sequences facilitates high-throughput and comprehensive description of bacteria

**DOI:** 10.1038/s43705-021-00017-z

**Published:** 2021-05-20

**Authors:** Thomas C. A. Hitch, Thomas Riedel, Aharon Oren, Jörg Overmann, Trevor D. Lawley, Thomas Clavel

**Affiliations:** 1 Functional Microbiome Research Group, RWTH University Hospital, Aachen, Germany; 2 Leibniz Institute DSMZ-German Collection of Microorganisms and Cell Cultures, Braunschweig, Germany; 3 German Center for Infection Research (DZIF), Partner site Hannover-Braunschweig, Braunschweig, Germany; 4 The Institute of Life Sciences, The Hebrew University of Jerusalem, The Edmond J. Safra Campus, Jerusalem, Israel; 5 Braunschweig University of Technology, Braunschweig, Germany; 6 Host-Microbiota Interactions Laboratory, Wellcome Sanger Institute, Hinxton, UK

**Keywords:** Bacteria, Phylogenetics, Phylogenomics

## Abstract

The study of microbial communities is hampered by the large fraction of still unknown bacteria. However, many of these species have been isolated, yet lack a validly published name or description. The validation of names for novel bacteria requires that the uniqueness of those taxa is demonstrated and their properties are described. The accepted format for this is the protologue, which can be time-consuming to create. Hence, many research fields in microbiology and biotechnology will greatly benefit from new approaches that reduce the workload and harmonise the generation of protologues.

We have developed Protologger, a bioinformatic tool that automatically generates all the necessary readouts for writing a detailed protologue. By producing multiple taxonomic outputs, functional features and ecological analysis using the 16S rRNA gene and genome sequences from a single species, the time needed to gather the information for describing novel taxa is substantially reduced. The usefulness of Protologger was demonstrated by using three published isolate collections to describe 34 novel taxa, encompassing 17 novel species and 17 novel genera, including the automatic generation of ecologically and functionally relevant names. We also highlight the need to utilise multiple taxonomic delineation methods, as while inconsistencies between each method occur, a combined approach provides robust placement. Protologger is open source; all scripts and datasets are available, along with a webserver at www.protologger.de

## Introduction

The recent renaissance of cultivation has led to >500 novel species being added to the ‘List of Prokaryotic names with Standing in Nomenclature’ (LPSN) database every year since 2005^1^.

This has included large-scale cultivation projects of host-associated microbial communities^2–9^ as well as environmental sources, such as soil^10^ and the ocean^11,12^. Whilst many novel isolates are being cultured in such studies, few are taxonomically described with names that are validly published. The lack of names prevents the quick and unique referencing of these taxa, hampering researchers’ ability to study these species further. This topic affects a wide range of specialities within microbiology, yet has not been addressed.

In addition to culture-dependent studies, metagenomic approaches can provide complementary results, increasing the ability to study a microbial community^2,13,14^. In particular, metagenome-reconstructed genomes (MAGs) are of increasing importance for studying the functions and taxonomy of prokaryotes, although without cultured representatives these functions cannot be validated experimentally. MAGs allow the study of as-yet-uncultured taxa, such as the entire phylum of “*Candidatus* Lokiarchaeota”^15^. Currently, MAGs cannot be utilised as type material for providing a validly published name, although ‘*Candidatus*’ taxa can be proposed. Although the rank of ‘*Candidatus*’ taxa is not formally included in the rules of the International Code of Nomenclature of Prokaryotes (ICNP)^16^, still a ‘protologue’ describing the taxon is required (Appendix 11 of the ICNP). In addition, MAGs provide an invaluable background of potentially novel taxa, on to which cultured isolates can be compared, strengthening the justification of creating high taxonomic groups, e.g., families^17^. One application of this method is GTDB-Tk^18^, a state-of-the-art resource which utilises the genomes of both isolates and MAGs to place queried genomes within the currently sequenced space of taxa. By expanding the taxonomic and genomic landscape, MAGs have facilitated detailed analysis of both described and undescribed taxonomic groups^19–21^.

When describing a novel taxon, few guidelines exist with rule 27.2.c of the ICNP stipulating that “The properties of the taxon being described must be given directly after [the name] and [its etymology]”^16^. This format is termed a protologue and acts as a standardised format for describing a novel taxon in a clear and concise manner^22^. Further recommended minimal descriptions have been published for specific lineages, however, these are only recommendations according to the ICNP^23–25^.

Single marker genes are a common element of protologues, including the 16S rRNA gene sequence. However, the advent of genome sequencing has seen a rise in genome-based measures of taxonomic diversity. These include gene content dissimilarity^26^, average nucleotide identity (ANI)^27^, average amino acid identity (AAI)^28^, digital DNA–DNA hybridisation (dDDH)^29^, percentage of conserved proteins (POCP)^30^, differences in the G + C content of genomic DNA^31^ and integration into large-scale phylogenetic trees^32,33^. In addition, protologues generally describe the functional and ecological niche of a given taxon.

Currently, no single tool provides users with the taxonomic, functional and ecological insights required for writing a protologue. For example, GTDB-Tk^18^, MiGA^34^ and TYGS^35^ taxonomically place a given genome, yet none of these methods provide functional or detailed ecological readouts. GTDB-Tk output is based on placement within a pre-calculated phylogenomic tree, generating a relative evolutionary distance (RED) value, for placement of novel lineages, and directing targeted ANI^27^ comparisons to existing closely related species. MiGA also utilises ANI, as well as AAI for placement and uniquely does not apply any tree-based methods. Similarly, TYGS generates a tree based on blast similarity^35–37^ and provides dDDH^29^ values to the close relatives. The lack of secondary or tertiary taxonomic assignment methods in these existing tools, limits the ability of the user to confirm the robustness of the taxonomic placement. In addition, the functional and ecological features of a taxon can also be used to supports its differentiation from close relatives. By providing seven lines of taxonomic evidence combined with ecological and functional readouts, users of Protologger obtain all the building blocks required for the description of novel, or known, taxa. This reduces the burden on the user and introduces consistency in the description of taxa.

In this paper, we introduce Protologger, an all-in-one tool that automatically describes the taxonomic, functional and ecological features of a species, providing output that can be used directly for writing a protologue.

## Results

### Protologger workflow

**Fig. 1:**
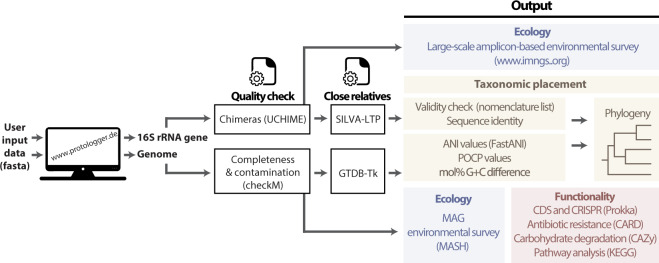
Simplified overview of Protologger. The key steps within Protologger are highlighted with the tools utilised for each step indicated (in brackets), along with the quality assurance steps. Sections are coloured according to the information they provide with taxonomic placement (in yellow), ecology (in blue), and functionality (in red). The ‘validity check’ stage in taxonomic assignment involves the removal of taxa without validly published names from genomic comparison.

Protologger requires the 16S rRNA gene and genome sequence of a single species and delivers multiple taxonomic, ecological, and functional readouts using specific databases and tools (Fig. 1). In addition, Protologger conducts quality checks on both the genome and 16S rRNA gene sequence, in line with the proposed guidelines for the use of genome sequences for taxonomic purposes^38^. A detailed description of all analysis steps can be found in the “Methods”.

**Fig. 2:**
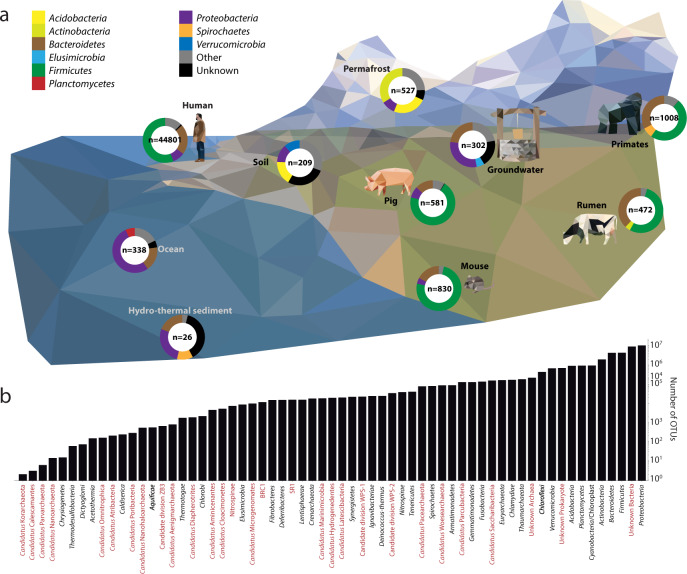
Overview of the ecological databases used within Protologger. **a** Representation of the diverse environments from which MAGs originate from in the ecological analysis. For each environment^13,14,39–46^, the number of MAGs is stated, along with a pie chart indicating the three most prevalent bacterial phyla (see colour code in the figure), as determined by GTDB-Tk cross-referenced with LPSN. MAGs termed as ‘generic’ due to a lack of metadata are not included (*n* = 3397)^47^. **b** Phylum level taxonomic diversity within the IMNGS amplicon studies utilised within the 16S rRNA gene amplicon-based habitat preference and distribution analysis. These datasets span 63 phyla represented by over 37,314,233 OTUs. The names of phyla lacking a child taxon with a validly published name are in red, as determined via the LPSN database.

In brief, taxonomic assignment is conducted via identification of the 50 closest relatives within the SILVA Living Tree Project based on 16S rRNA gene sequence identity. Species with validly published names according to the DSMZ nomenclature list, supplemented with updates from LPSN, have their type genomes obtained from the GTDB database and used to calculate genome-based delineation values: ANI, POCP, and differences in the G + C content of genomic DNA. Species lacking valid names are discarded from genomic analysis due to the lack of standing within the ICNP. In addition, genomes are assigned a taxonomic lineage using GTDB-Tk^18,33^. Functional analysis is conducted using the proteome predicted from the genome sequence file, annotated against KEGG for pathway analysis, CAZy for carbohydrate metabolism, and CARD for profiling the species antibiotic resistance. Ecological analysis is conducted using both the 16S rRNA gene sequence, as well as the genome. First, the genome is compared to an internal collection of >49,000 metagenome-assembled genomes (MAGs) (Fig. 2a) collected from across ten different environments^13,14,39–47^. Second, ecological occurrence is calculated by comparing the 16S rRNA gene sequence to operational taxonomic units (OTUs) generated from 19,000 amplicon datasets (1000 from each of 19 environments, defined in the methods). The taxonomic distribution of these OTUs highlights the inclusion of many unknown taxa, although as a whole, the database is dominated by *Proteobacteria* (Fig. 2b).

Protologger is entirely open source, hence the code and databases can be accessed via the GitHub repository (github.com/thh32/Protologger) and a dedicated Galaxy-based website is available (www.protologger.de), which includes an instructional video. For local installation, ~100 Gb RAM and ~200 Gb storage space are required due to the integration of GTDB-Tk and its associated databases.

### Comparison of taxonomic delineation methods within large-scale collections of gut bacterial isolates

Three recently published, large-scale collections of isolates were combined to provide a diverse dataset on which to compare the taxonomic delineation readouts provided by Protologger, including 16S rRNA gene sequence similarity, ANI, POCP, and GTDB-Tk assignment. From the initially reported number of 737 isolates within the human bacterial collection (HBC)^6^, 3632 within the Broad Institute-OpenBiome Microbiome Library (BIO-ML)^7^, and 410 within the Hungate1000 isolate collection^3^, dereplication (>95% ANI) led to the identification of 435, 206 and 308 species-level genomes, respectively, which were analysed further. Of these (*n* = 949), complete analysis and output were provided for 851 (HBC: 422, BIO-ML: 197, Hungate1000: 232); the failure of some genomes to be analysed was due to the inability to identify and extract a 16S rRNA gene sequence from the isolates’ genome, which was conducted before input.

**Fig. 3:**
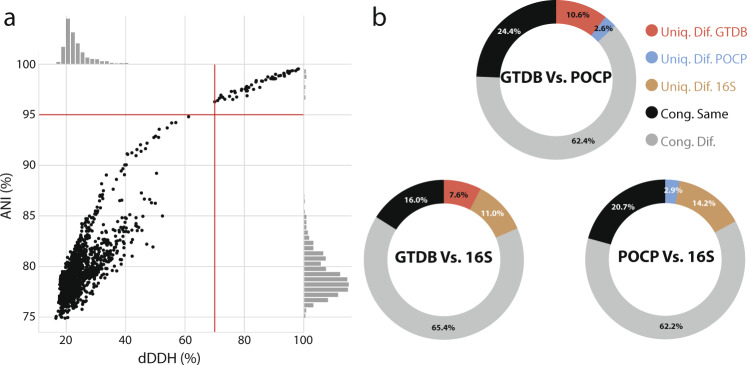
Comparison of taxonomic delineation methods. **a** Pairwise comparisons between the dDDH and ANI values obtained for 70 isolates and their closest relatives, as identified by Protologger (*n* = 1599). Red lines highlight published boundaries for species delineation: dDDH, <70%; ANI, <95%. **b** Pairwise comparisons to test the consistency of genus-level delineation parameters (*n* = 30,247). For each comparison, four groups were formed based on how each method assigned the paired genomes: congruent results, same genus (Cong. Same), congruent results, different genera (Cong. Dif.) and those uniquely identified as belonging to different genera according to the method specified (Uniq. Dif.). See colour code in the figure.

For many years, DDH has been the gold standard for delineation of bacterial species. However, due to the difficulty in applying this method experimentally, bioinformatic proxies have been developed including dDDH^29^ and ANI^48^. Initial experiments confirmed that ANI values strongly correlate with those from DDH experiments^48^ and large-scale analysis of genomic data has set the current threshold for species delineation using ANI at >95%^27^. Nonetheless, to validate the use of FastANI^27^ within Protologger, we compared the consistency of both FastANI and dDDH to delineate genomes belonging to the same, or different species. For this, isolates for which Protologger predicted species matches were randomly selected from the three isolate collections (*n* = 70 isolates). The pairwise genome comparisons for each isolate (*n* = 1599) were run through FastANI and the same genomes uploaded to the GGDC server to obtain dDDH values^37^, as no open-source version is available preventing large-scale comparison (Fig. 3a). We observed 100% consistency in both methods’ delineation of species, along with a strong positive correlation between the scores (Pearson *R*^2^ = 0.92, *P* < 0.01). These data support the use of FastANI values for species-level delineation, ensuring the open-source nature of this project.

For genus-level delineation, Protologger provides three suitable readouts: POCP, GTDB assignment, and 16S rRNA gene sequence similarity (Fig. 1). Using each methods’ results from the pairwise comparison of isolates from the three collections (*n* = 834) to their closest relatives (*n* < 50 per isolate), those for which both pairwise genome and 16S rRNA gene data was present were extracted, resulting in 30,247 pairwise comparisons. The consistency of the three methods to assign these comparisons as either intra- or inter-genus was compared (Fig. 3b). The methods were >80% congruent when compared pairwise, and even when all compared simultaneously, 75.6% congruence was observed. 16S rRNA gene sequence similarity showed a relatively high degree of congruence with GTDB (81.4%) and POCP (82.9%) (Fig. 3b), although it uniquely assigned >10% of the pairings as originating from different genera. The majority of these comparisons were inter-family, as determined congruently between GTDB assignments and 16S rRNA gene sequence similarity scores (Supplementary Fig. 1), with only 11.3% of the comparisons occurring between members of different families. This is due to these comparisons being based on Protologger output which limits the comparisons to the 50 closest relatives. This confirms the need to integrate multiple lines of evidence during taxonomic assignment to guide the placement of a novel taxon.

### Description of taxonomic novelty

**Fig. 4:**
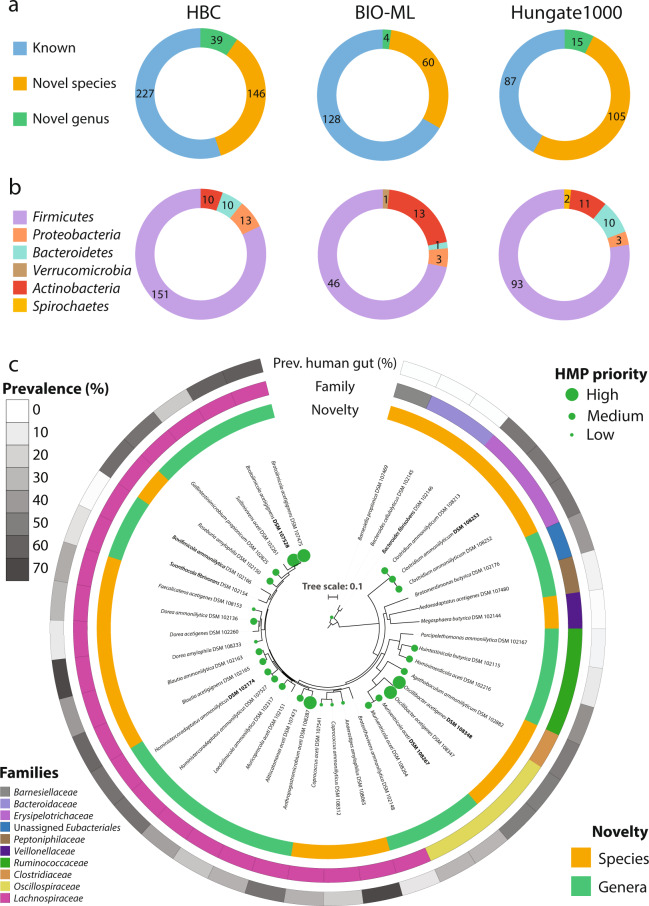
Uncovering and describing taxonomic novelty using Protologger. All non-redundant species-level isolates from three large collections were processed: the human bacterial collection (HBC)^6^, the Broad Institute-OpenBiome Microbiome Library (BIO-ML)^7^ and the Hungate1000 collection^3^. **a** Each collection contained novel taxa, representing either undescribed species or genera. **b** Phylum level diversity of the undescribed isolates. **c** Phylogenomic tree of the novel HBC isolates described and named. For some species, multiple strains were identified; therefore, the type strain DSM number is in bold (see protologues). Isolates matching HMP ‘most wanted’ species are highlighted with green balls at the branch tips with the size representing priority. The external rings represent isolate specific information as follows: (i) the inner ring highlights the novelty, either species or genus; (ii) the centre ring indicates to which family the isolates are assigned; (iii) the outer ring shows the prevalence of each isolate across 1000 human gut amplicon samples (the ecosystem of origin of the isolates), with values ranging from 1.0–69.6%.

Each of the isolate collections originating from the gastrointestinal tract was identified to contain multiple novel taxa: 311 novel species and 58 novel genera (Fig. 4a). The majority belonged to the phylum *Firmicutes*, although novel members of the *Bacteroidetes*, *Actinobacteria* and *Proteobacteria* were also present (Fig. 4b). As many of the isolates within the HBC collection^6^ were made publicly available via deposition at national reference collections, we utilised Protologger to taxonomically describe and provide validly published names for them. Out of the 72 isolates, we deposited 40 at a second national culture collection, which is mandatory for the valid publication of names. These 40 isolates represented 34 novel taxa across 9 families, including 17 novel species and 17 novel genera (Fig. 4c).

To identify whether these isolates represent species previously identified as being of importance within the human gut microbiome, we compared the isolates’ 16S rRNA gene sequences to the OTU sequences of the Human Microbiome Projects (HMP) ‘most wanted’ taxa^49^. Within the ‘most wanted’ list, taxa were stratified into three priority levels; low, medium, and high based on their perceived novelty when compared to human-specific strain collections. Out of the 34 novel taxa described here, HMP ‘most wanted’ species matches were identified for 22, including 3 high-, 13 medium- and 6 low-priority OTUs (Fig. 4c).

Using the habitat preference and distribution analysis conducted by Protologger (Figs. 1 and 2), we were able to better understand the importance of these newly named bacteria across multiple ecosystems. For example, strain Sanger_90 was most commonly identified within pig gut (26.3%) and human gut (7.4%) samples, although sub-dominant in both environments at 0.1% and 0.2% mean relative abundance, respectively. Due to the integration of the ‘Great Autonomic Nomenclator’ (GAN)^50^ within Protologger, the novel genus represented by this strain Sanger_90 was named ‘*Porcipelethomonas’*, due to being most commonly present within pig gut samples. Similarly, this occurred with the medium level HMP ‘most wanted’ species, ‘*Laedolimicola ammoniilytica’* and ‘*Huintestinicola butyrica’*, were named due to being present in 65.7% of chicken gut samples and 29.2% of pig samples, respectively. Observations such as these are not possible with targeted analysis of individual environments. All 34 novel taxa described within this work were named according to this method and protologues are provided at the end of the methods section.

### High-quality MAGs support the study of as-yet-uncultured taxonomic lineages

MAGs represent an invaluable resource for both the phylogenetic placement and the functional study of isolates within understudied lineages. Currently, MAGs cannot be utilised to describe novel taxa with validly published names, although ‘*Candidatus*’ taxa can be proposed according to the rules of the ICNP.

An alternative ﻿nomenclatural code for prokaryotes has been recently proposed, called the International Code of Nomenclature of Uncultivated Prokaryotes (ICNUP), aiming at valid publication of names of as-yet-uncultured taxa using their genomic information as the type material^51^. Hence, the creation of detailed protologues with help of Protologger is also relevant in the context of cultivation-independent taxonomy.

**Fig. 5:**
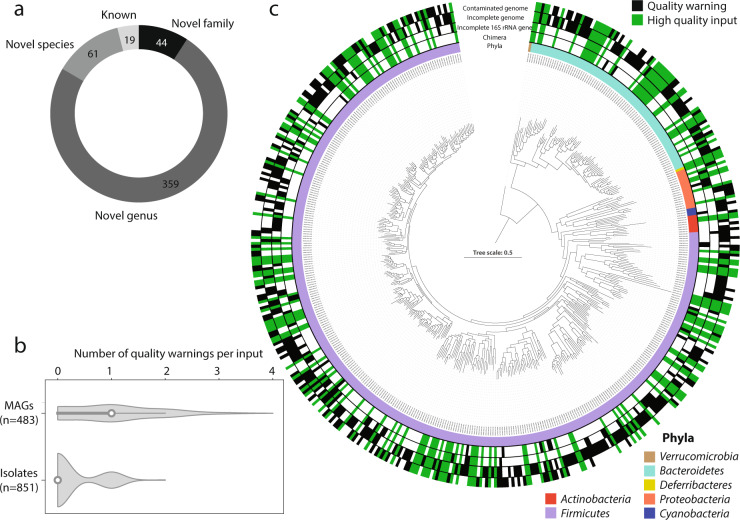
Quality and novelty within a MAG dataset from the mouse intestine^43^. **a** Novelty of the 484 iMGMC MAGs according to their 16S rRNA gene sequence similarity to their closest relative. **b** Comparison of the MAG and isolate collections error warnings per input generated by Protologger. White dots show the median number of errors while the dark grey bar highlights the interquartile range and the black line indicates the lower/upper adjacent values. **c** Phylogenomic tree of all 484 MAGs with rings on the outside highlighting in black the occurrence of Protologger warnings: chimeric 16S rRNA gene sequences, incomplete 16S rRNA gene sequences, incomplete genomes, contaminated genomes. The MAGs with no warning, hence deemed of high quality, are indicated by green bars.

The development of bioinformatic methods to link MAGs to 16S rRNA gene sequences facilitates their use as input into Protologger^43^. This inclusion of 16S rRNA gene sequences is essential due to the lack of genomes for many described prokaryotes with validly published names. With the potential of such data being used to describe and name novel taxa as ‘*Candidatus*’, we aimed to assess the ability of Protologger to provide reliable information. Currently, the system analyses the quality of input data for detection of chimeric 16S rRNA gene sequences, incomplete 16S rRNA gene sequences (<80%), contaminated genomes (>3%), and incomplete genomes (<95%). Analysis was conducted on the iMGMC dataset^43^, which consists of 484 MAGs from the mouse intestine that were matched to 16S rRNA gene sequences using a combination of annotation and co-occurrence analysis^43^. The iMGMC dataset was dominated (96%, 465 MAGs) by representatives of novel taxa based on the ANI, POCP and GTDB-Tk assignment, including 44 representatives of novel families (Fig. 5a). Overall, warnings about the quality of the MAGs were produced in 67% of cases, in comparison to 28.9% across all three isolate collections (Fig. 5b). The quality of the genomes included within the MAG dataset was highly variable with 39.5% being deemed ‘high-quality’ (>95% complete, <3% contamination), producing no warning. In addition to the genome quality warning, 21 instances of chimeric 16S rRNA gene sequences and 71 incomplete 16S rRNA genes were detected (Fig. 5c). The ubiquitous nature of these quality issues across the dataset suggests that they are not linked to specific lineages but inherent to this method. Hence, users with such data should be aware during analysis. In the event of the ICNUP being established, the Protologger output for all 484 MAGs has been made available (see “Methods”), facilitating the description and naming of these novel taxa.

## Discussion

The renewed interest in cultivation, as well as the use of metagenomic datasets to infer the existence of novel bacterial lineages highlights the dire need for automated description and naming of novel taxa^50,51^. Protologger aims to facilitate this process by providing all-important building blocks for users to study the taxonomic placement, ecological occurrence, and main functional characteristics of their taxon of interest.

By providing users with seven lines of taxonomic information based on both 16S rRNA gene sequence and genome data, users can easily integrate the provided information to decide the final placement of their taxon. Published thresholds of the delineation methods utilised in Protologger were used to compare the ability of each method to differentiate novel from known species and genera. While consistent in most cases, differences occurred, further highlighting the importance of providing multiple lines of evidence as done in Protologger. For example, although POCP values are cited for all novel genera proposed in this paper, many fell within the range of 45–55%, highlighting the need for manual evaluation of borderline values in light of all the taxonomic output provided. Altogether, we strongly recommend an educated examination of all parameters prior to making a final decision on the placement of novel taxa. Whilst this may represent a burden on the user, it is imperative to ensure robust assignment within the existing taxonomy. As phylogenetic and phylogenomic trees can further support the placement of novel taxa beyond threshold values, both types of trees are also provided by Protologger. While physiological and phenotypic features are predicted, the expression and utilisation of these pathways cannot be guaranteed, hence observation of the species to validate the physiological features as well as phenotypic testing are recommended to verify the predictions.

In our hands, Protologger significantly reduces the manual workload required in protologue generation from ~10 h to ~1 h. This was shown via application to four isolate- and MAG-based datasets, facilitating the description of 34 novel taxa from the human gut, including 22 previously reported to be of particular interest to the community. By simplifying the process of describing taxa, we believe that the names of a greater number of new taxa will become validly published in the future. The naming of bacteria has also been highlighted as an area of difficulty due to the need for knowledge of Latin and Greek^52^. The integration of Protologger’s overview file with the ‘GAN’^50^ allows the creation of names derived from the characteristics of the isolate being studied, preventing names based on taxonomic relatedness to existing taxa with validly published names or the use of placeholder identifiers. By removing these barriers, we make the description and naming of novel taxa a reality for researchers who may have dismissed the idea previously due to the additional workload it represents. Continual development of the system is underway to make these descriptions can available to the wider research community via integration with BacDive. Users are able to email the Protologger overview file to BacDive (contact@bacdive.de), along with the BacDive identifier of the studied isolate, and the two will be linked. This will ensure the longevity of the output and facilitate citable species descriptions as BacDive entries have digital object identifier numbers. Furthermore, this will facilitate transparent discussion on the taxonomic placement of taxa as all delineation values will be available to the community.

Issues regarding data quality were observed within both isolate- and MAG-based datasets, suggesting users must be aware of such issues regardless of the input used. In this age, when taxa can be described purely based on their genome^53^, minimum quality thresholds are essential to prevent erroneous findings. Such minimum thresholds have previously been proposed, but have yet to be made mandatory^38,51^. We therefore re-state the need for defined and enforced minimum genome standards for the description of novel taxa within the existing ICNP as well as in the context of the ICNUP^51^.

Whilst reliant on external databases, Protologger will be updated according to each release of both the Living Tree Project and the GTDB database, ensuring its continual relevance to the community. Furthermore, the complete open-source nature of Protologger, including its dependencies, ensures that all researchers can access this resource. The identification that fastANI provides equally reliable species-level delineation as dDDH was essential for this, to prevent the reliance on closed source tools. The additional development and maintenance of a webserver (www.protologger.de) aims to facilitate researchers without either bioinformatic skills or the resources necessary for Protologger to have access to this tool. As a tool designed for the community, we welcome researchers to contribute to the continual development, such as via the proposal of additional pathways or habitats of interest.

## Materials and methods

### Input

Protologger requires both the full-length 16S rRNA gene sequence and genome assembly of an isolate to be submitted to the system as FASTA files (Fig. 1). As described below, the 16S rRNA gene sequence is then used for the taxonomic placement of the isolate whilst the genome reconfirms the placement, along with providing insight into the functional repertoire of the isolate (described below in ‘Functional analysis’). Both are also used for the generation of ecological readouts.

### Taxonomic assignment

Valid publication of names of novel taxa relies on taxonomic comparison of an isolate to closely related species with a validly published name. The use of 16S rRNA gene sequences to delineate taxa goes back to the 1970s when such sequences were used to provide the first evidence that the Archaea and Bacteria represented distinct lineages of Prokaryotes^54^. For this, Protologger compares input 16S rRNA gene sequences to the Living Tree Project (LTP)^55^ (LTPs132), a subsidiary of the SILVA database^56^, which is regularly updated and consists of representative 16S rRNA gene sequences from all isolated taxa.

The quality of the input 16S rRNA gene sequence is checked via detection of chimeras using UCHIME (Usearch v5.2.32)^57^ with the LTP database as a reference. BLASTN (v2.10.0+) (>60% identity, >80% query coverage and evalue <10^−25^) is used to identify the 50 highest scoring species in the LTP database and pairwise sequence similarity is calculated^58^. Delineation of taxa based on 16S rRNA gene sequence similarity currently stands at 98.7% for species, 94.5% for genera, and 86.5% for families^59^. However, some taxonomic lineages require the use of altered delineation thresholds due to their unique genomic makeup or evolutionary history^60,61^; hence all values are provided to the user. The quality of the provided 16S rRNA gene sequence is checked by comparison against its closest match, providing a completeness value, which is reported to the user. These sequences are then aligned using MUSCLE^62^ (v3.8.31), default settings, and further processed using FastTree^63^ (v2.1.7), GTR model, to generate a 16S rRNA gene sequence-based phylogenetic tree.

Genomes are firstly checked for completeness and contamination using CheckM^64^ (v1.0.12). Concatenated protein sequence trees using multiple marker genes have been extensively applied for the phylogenetic placement of genomes^32,33^. The Genome Taxonomy DataBase (GTDB) is currently the most exhaustive approach to tree-based genome taxonomy, utilising 120 bacterial marker genes to determine the REDs between ~100,000 genomes, facilitating standardised distances for each taxonomic level^33^. Protologger uses GTDB-Tk (r89) (v1.2.0) for the placement within the GTDB taxonomy system via the detection of marker genes to assign a domain. These marker genes are then used to place the genome within the domain-specific reference tree and ANI values confirm the species-level identification^18^.

The genomes of closely related species, as determined by 16S rRNA gene sequence similarity scores, are obtained from the GTDB type genome list. Before their inclusion in downstream analysis, only those with validly published names are accepted. This is checked by comparison against those taxa with validly published named within the ‘List of Prokaryotes with Standing in Nomenclature’ maintained by the Leibniz Institute DSMZ-German Collection of Microorganisms and Cell Cultures^1^.

Once the list of close relatives has been populated, the percentage (mol%) of guanine + cytosine (G + C) of the genomic DNA, ANI and POCP values are calculated pairwise against the input genome. Protologger utilises FastANI (v1.2) to calculate ANI values^27^ and custom python scripts for POCP calculation, available in the GitHub repository.

Previously, it was assumed that the high variability in mol% G + C between isolates belonging to the same species, up to 5%^65^, prevented its use for species delineation^66^. However, reanalysis suggested these variations were due to methodological issues that do not exist when genome sequences are used to calculated G + C content and that genomes from the same species rarely vary by >1%^31^. Protologger contains custom scripts, provided in the GitHub repository, for calculating G + C (%).

The POCP value calculated between the genomes of two strains has also been proposed for delineation of genera based on values <50%^30^. This threshold has since been used within the *Chlamydiales*^67^, *Pseudomonas*^68^ and *Muribaculaceae*^17^. Protologger calculates POCP values according to the original publication^30^.

### Functional analysis

Protologger provides detailed analysis of the functional repertoire of input genomes including pathway analysis (KEGG)^69^, carbohydrate activate enzyme (CAZy)^70^ and detection of antibiotic resistance genes (CARD)^71^.

Proteins are first extracted from both the query genome and the reference genomes, facilitating calculation of POCP values, using PROKKA^72^ (v1.14.6) (default parameters), during which the number of CRISPR arrays within the genome are recorded. KEGG orthologs (KOs) are determined using the PROKKA output, providing a non-redundant list of KOs via PROKKA2KEGG (https://github.com/SilentGene/Bio-py/tree/master/prokka2kegg). These KOs are then compared to manually selected pathways including utilisation of 10 carbon sources (glucose, arbutin, salicin, cellobiose, sucrose, trehalose, maltose, starch, dextran, cellulose), short-chain fatty acid biosynthesis (acetate, propanoate/propionate, butanoate/butyrate), siroheme biosynthesis, urease, vitamin biosynthesis (biotin (vitamin B7), cobalamin (vitamin B12), folate (vitamin B9), riboflavin (vitamin B2), menaquinone (vitamin K2) and phylloquinone (vitamin K1)), EPS biosynthesis, cytochrome c oxidase, ammonia production, nitrification, ammonia utilisation, sulfide utilisation and sulfate assimilatory reduction. Genes encoding additional features, such as the presence of flagella-encoding genes (*n* = 25) and spore production (*n* = 52) are also studied. These pathways were selected due to either representing an inherent feature of the species *e.g*. spore formation or motility, or being key to their role in their environment *e.g*. nitrogen fixation, vitamin biosynthesis. Currently, 30 different biochemical and physiological features are assessed; however, this will continue to be updated.

### Habitat preference and distribution

The 16S rRNA gene amplicon-based habitat preference and distribution analysis are determined using a comprehensive database of 19,000 16S rRNA amplicon samples from 19 environments obtained from the IMNGS database^73^ and representing a total of 38,163,501 OTUs (Fig. 2b). These environments are activated sludge, bovine gut, chicken gut, coral, freshwater, human gut, human lung, human oral, human skin, human vagina, insect gut, marine, marine sediment, mouse gut, pig gut, plant, rhizosphere, soil and wastewater. Comparison of the query 16S rRNA sequence to this database is done via BLASTN (97% identity, 80% OTU coverage).

In addition, input genomes are compared to a database of 49,094 high-quality (>90% complete, <5% contamination), based on published thresholds^74^, MAGs obtained from 12 studies and at least 10 environments (Fig. 2a). The comparison is conducted using MASH^75^ (v2.2), with results filtered using a distance threshold of <0.05. Comparison against the MAG database provides supporting information for the identification of novel taxonomic groups, reducing the reliance on descriptions from single isolates.

### Webserver

Protologger is available via a custom Galaxy installation, hosted at the University Hospital of RWTH Aachen, at www.protologger.de. The website contains detailed instructions, as well as an instructional video to guide users.

### Datasets

Protologger was applied to three large isolate collections which focused on the gastrointestinal tract. The first collection, Forster et al.^6^, consists of 737 isolates, of which strains representing new taxa were deposited at various national culture collections and their genomes were sequenced. The second collection, Poyet et al.^7^, consists of 7758 isolates, of which 3632 had been genome sequenced. Isolates from this collection are maintained locally, forming the BIO-ML culture collection, but not submitted to public culture collections^7^. The Hungate1000 collection is formed from 410 cultured bacteria and archaea for which genomes are available, yet few strains were deposited to public culture collections. All Archaeal genomes were ignored during downstream analysis due to Protologger being Bacteria specific. While they cannot be used for validation of names of novel taxa, MAGs can provide greater insight into currently as-yet-uncultured lineages. Therefore, a representative dataset of MAGs was selected for input to both tests the quality of the MAGs and highlight the use of Protologger to describe the novel taxa. For this, the integrated mouse gene catalogue (iMGMC)^43^ was used as it represents an advancement in the generation of 484 MAGs which were linked to full-length 16S rRNA gene sequences using a mixture of sequence mapping and correlation analysis.

The three isolate collections lacked 16S rRNA gene sequences for the corresponding strains, hence Barrnap was used to identify the presence of 16S rRNA genes within the genome sequences^72^. The longest 16S rRNA gene sequence identified for each genome was used as input for Protologger along with the original genome. The output for all analysed genomes is provided at github.com/thh32/Protologger.

### Protologues

All taxonomic, functional and ecological features used to describe novel taxa below are solely based on the output of Protologger. The names proposed were produced using the GAN^50^, modified to accept Protologger output. For novel genera, the occurrence of the given taxon in which environment is calculated as the prevalence x mean relative abundance in positive samples. The environment identified then directs GAN to utilise one of the curated lists of environmental prefixes, producing ecologically informed names. Species names are selected based on the isolate’s functional repertoire. This modified version of GAN is available at www.protologger.de. The Protologger output for all isolates described below are provided at github.com/thh32/Protologger.

#### Description of *Aedoeadaptatus* gen. nov

*Aedoeadaptatus* (Gr. neut. n. *aidoion*, the female pudendum; L. past. part. *adaptatus* adapted; N.L. masc. n. *Aedoeadaptatus*, a microbe frequently occurring in the female reproductive tract). This taxon is named referring to the microbial ecosystem with combined prevalence and mean relative abundance for this taxon, although originally isolated from human faeces. Based on 16S rRNA gene sequence similarity, the closest relatives are *Peptoniphilus* (*Peptoniphilus methioninivorax*, 88.5%; *Peptoniphilus stercorisuis*, 88.1%; *Peptoniphilus asaccharolyticus*^T^, 87.3%) and *Tissierella* (*Tissierella creatinini*, 87.7%; *Tissierella praeacuta*^T^, 86.3%). POCP analysis confirmed that strain E51 represents a distinct genus to *Peptoniphilus* (*P. asaccharolyticus*^T^, 44.0%) and *Tissierella* (*T. praeacuta*^T^, 30.0%) as all POCP values to close relatives were below 50%. GTDB-Tk supported the creation of a novel genus, placing strain E51 within the genus ‘Peptoniphilus_C’, a sub-division of the existing *Peptoniphilus* genus. The type species of this genus is *Aedoeadaptatus acetigenes*.

#### Description of *Aedoeadaptatus acetigenes* sp. nov

*Aedoeadaptatus acetigenes* (L. neut. n. *acetum*, vinegar; Gr. v. *gennaô*, to produce; N.L. part. adj. *acetigenes*, producing acetate). KEGG analysis identified pathways for acetate production from acetyl-CoA (EC:2.3.1.8, 2.7.2.1), propionate production from propanoyl-CoA (EC:2.3.1.8, 2.7.2.1), sulfide and l-serine utilised to produce l-cysteine and acetate (EC:2.3.1.30, 2.5.1.47), l-glutamate production from ammonia via l-glutamine (EC:6.3.1.2, 1.4.1.-) and folate (vitamin B9) biosynthesis from 7,8-dihydrofolate (EC:1.5.1.3). This species was commonly identified within human vaginal (24.2%) and human gut (8.2%) samples, although sub-dominant in both environments at ca. 0.2% mean relative abundance. The type strain, E51^T^ (=DSM 107480^T^), was isolated from human faeces. The G + C content of genomic DNA is 48.9%.

#### Description of *Agathobaculum ammoniilyticum* sp. nov

*Agathobaculum ammoniilyticum* (N.L. neut. n. *ammonium*, ammonia; N.L. neut. adj. *lyticum*, able to loose, able to dissolve; from Gr. neut. adj. *lytikon*, able to loose, dissolving; N.L. neut. adj. *ammoniilyticum*, ammonia-degrading, to reflect the activity of the bacterium). The species was identified as a member of the genus *Agathobaculum*, based on 16S rRNA gene sequence similarity of 96.8% and 96.7% to the existing members of the genus, *Agathobaculum butyriciproducens* and *Agathobaculum desmolans*. This was supported by POCP values of 56.7 and 63.4% to each member, respectively. ANI values to each of these related species were below 95%, which was confirmed by GTDB-Tk identification as ‘Agathobaculum-sp900291975’. Within the genome, 137 CAZymes were identified, facilitating the predicted utilisation of both starch and glucose as carbon sources. KEGG analysis identified a total of 93 transporters, 7 secretion genes and 513 enzymes. This included pathways for acetate production from acetyl-CoA (EC:2.3.1.8, 2.7.2.1), propionate production from propanoyl-CoA (EC:2.3.1.8, 2.7.2.1), riboflavin (vitamin B2) biosynthesis from GTP identified (EC:3.5.4.25, 3.5.4.26, 1.1.1.193, 3.1.3.104, 4.1.99.12, 2.5.1.78, 2.5.1.9, 2.7.1.26, 2.7.7.2) and l-glutamate production from ammonia was identified via l-glutamine (EC:6.3.1.2, 1.4.1.-). The type strain, Sanger_34^T^ (=DSM 102882^T^), was isolated from human faeces. The G + C content of genomic DNA is 57.0%.

#### Description of *Alitiscatomonas* gen. nov

*Alitiscatomonas* (L. masc./fem. n. *ales*, a bird; Gr. neut. n. *skor*, dung; L. fem. n. *monas*, a monad; *Alitiscatomonas*, a microbe frequently occurring in the faeces of birds). This taxon is named referring to the microbial ecosystem with combined prevalence and mean relative abundance for this taxon, although originally isolated from human faeces. Based on 16S rRNA gene sequence similarity, the closest relatives are members of the genus *Lacrimispora*: *Lacrimispora xylanolytica* (95.7%), *Lacrimispora aerotolerans* (95.5%) and *Lacrimispora sphenoides*^T^ (95.0%). POCP analysis confirmed that strain f_CCE represents a distinct genus to *Lacrimispora* as POCP analysis to all closest relative produced values below 50%, including to *L. sphenoides*^T^ (38.2%). GTDB-Tk supported the placement of strain f_CCE within a novel genus predicted metagenomically as ‘CAG-81’. The type species of this genus is *Alitiscatomonas aceti*.

#### Description of *Alitiscatomonas aceti* sp. nov

*Alitiscatomonas aceti* (L. neut. n. *acetum*, vinegar; L. gen. neut. n. *aceti*, of vinegar). The number of CAZymes identified within the type strains genome was 148. Strain f_CCE was predicted to utilise starch. KEGG analysis identified pathways for acetate production from acetyl-CoA (EC:2.3.1.8, 2.7.2.1), propionate production from propanoyl-CoA (EC:2.3.1.8, 2.7.2.1), l-glutamate production from ammonia via l-glutamine (EC:6.3.1.2, 1.4.1.-) and folate (vitamin B9) biosynthesis from 7,8-dihydrofolate (EC:1.5.1.3). This species was most commonly identified within chicken gut samples (56.8%), although sub-dominant at only 0.26% mean relative abundance. The type strain, f_CCE^T^ (=DSM 107473^T^), was isolated from human faeces. The G + C content of genomic DNA is 51.8%.

#### Description of *Anaerostipes amylophilus* sp. nov

*Anaerostipes amylophilus* (Gr. neut. n. *amylon*, starch; N.L. masc. adj. *philus* (from Gr. masc. adj. *philos*) loving; N.L. masc. adj. *amylophilus*, starch-loving). The isolate was identified as a member of the genus *Anaerostipes*. The comparison of 16S rRNA gene sequence to existing members of this genus identified a 99.3% match to *Anaerostipes hadrus* and 93.3% to the type species of the genus, *Anaerostipes caccae*^T^. The assignment of the isolate to *Anaerostipes* was supported by POCP values of 77.5% and 55.9% to *A. hadrus* and *A. caccae*, respectively. GTDB-Tk supported the placement within *Anaerostipes* and the generation of a novel species as the isolate was assigned to ‘Anaerostipes hadrus_A’. ANI comparison provided a value of 88.8% to *A. hadrus*, confirming they are not the same species, but share a conserved 16S rRNA gene sequence. Within the genome, 142 CAZymes were identified along with the utilisation of glucose, cellobiose and starch. KEGG-based analysis identified the presence of the following pathways: acetate production identified from acetyl-CoA (EC:2.3.1.8, 2.7.2.1), butyrate production from butanoyl-CoA (EC:2.8.3.8), propionate production from propanoyl-CoA (EC:2.3.1.8, 2.7.2.1), sulfide and l-serine utilised to produce l-cysteine and acetate (EC:2.3.1.30, 2.5.1.47), l-glutamate production from ammonia via l-glutamine (EC:6.3.1.2, 1.4.1.-) and folate (vitamin B9) biosynthesis from 7,8-dihydrofolate (EC:1.5.1.3). The type strain, H1_26^T^ ( = DSM 108065^T^), was isolated from human faeces. The G + C content of genomic DNA is 36.6%.

#### Description of *Anthropogastromicrobium* gen. nov

*Anthropogastromicrobium* (Gr. masc. n. *anthropos*, a human being; Gr. fem. n. *gaster*, the stomach; L. neut. n. *microbium*, a microbe; *Anthropogastromicrobium*, a microbe from the stomach of humans). Based on 16S rRNA gene sequence similarity, the closest relatives are members of the genera *Lacrimispora* (*Lacrimispora amygdalina*, 90.8%), *Lachnospira* (*Lachnospira multipara*^T^, 90.8%) and *Cuneatibacter* (*Cuneatibacter caecimuris*^T^, 90.7%). POCP analysis confirmed strain H6_35 represents a distinct genus to both *Lacrimispora and Lachnospira* as all POCP values to close relatives were below 50%. GTDB-Tk supported the creation of a novel genus, placing strain H6_35 within the predicted genus ‘KLE1615’. The type species of this genus is *Anthropogastromicrobium aceti*.

#### Description of *Anthropogastromicrobium aceti* sp. nov

*Anthropogastromicrobium aceti* (L. neut. n. *acetum*, vinegar; L. gen. neut. n. *aceti*, of vinegar). The number of CAZymes identified within the type strains genome was 292, facilitating the predicted utilisation of cellulose and starch. KEGG analysis identified pathways for acetate production from acetyl-CoA (EC:2.3.1.8, 2.7.2.1), butyrate production from butanoyl-CoA (EC:2.8.3.8), propionate production from propanoyl-CoA (EC:2.3.1.8, 2.7.2.1), sulfide and l-serine utilised to produce L-cysteine and acetate (EC:2.3.1.30, 2.5.1.47), l-glutamate production from ammonia via l-glutamine (EC:6.3.1.2, 1.4.1.-) and folate (vitamin B9) biosynthesis from 7,8-dihydrofolate (EC:1.5.1.3). The type strain, H6_35^T^ (=DSM 108287^T^), was isolated from human faeces. The G + C content of genomic DNA is 41.3%.

#### Description of *Bacteroides cellulolyticus* sp. nov

*Bacteroides cellulolyticus* (N.L. neut. n. *cellulosum*, cellulose; N.L. masc. adj. *lyticus*, able to loose, able to dissolve; from Gr. masc. adj. *lytikos*, dissolving; N.L. masc. adj. *cellulolyticus*, cellulose-dissolving). The species was identified as a member of the genus *Bacteroides*. The comparison of 16S rRNA gene sequence identified the highest matches to existing members of *Bacteroides*, including *Bacteroides caecigallinarum* (97.5%), although the similarity to the type species, *Bacteroides fragilis*, was only 90.5%. POCP analysis also confirmed the placement of strain Sanger_22 within the *Bacteroides* with values >50% to 26 existing *Bacteroides* species, although the value to *B. fragilis* was 47.4%. GTDB-Tk supported the placement of strain Sanger_22 within the *Bacteroides*, placed as ‘Bacteroides_A sp900066445’. ANI values to all close relatives were below 95%, confirming this isolate represents a novel species. Within the genome, 357 CAZymes were identified along with the utilisation of starch and cellulose. KEGG-based analysis identified the presence of the following pathways: acetate production from acetyl-CoA (EC:2.3.1.8, 2.7.2.1), propionate production from propanoyl-CoA (EC:2.3.1.8, 2.7.2.1), l-glutamate production from ammonia via l-glutamine (EC:6.3.1.2, 1.4.1.-), folate (vitamin B9) biosynthesis from 7,8-dihydrofolate (EC:1.5.1.3) and riboflavin (vitamin B2) biosynthesis from GTP (EC:3.5.4.25, 3.5.4.26, 1.1.1.193, 3.1.3.104, 4.1.99.12, 2.5.1.78, 2.5.1.9, 2.7.1.26, 2.7.7.2). The type strain, Sanger_22^T^ (=DSM 102145^T^), was isolated from human faeces. The G + C content of genomic DNA is 40.2%.

#### Description of *Barnesiella propionica* sp. nov

*Barnesiella propionica* (N.L. neut. n. *acidum propionicum*, propionic acid; L. suff. -*ica*, suffix used with the sense of pertaining to; N.L. fem. adj. *propionica*, pertaining to propionic acid). The species was identified as a member of the genus *Barnesiella*. The comparison of 16S rRNA gene sequence identified the highest matches to both existing members of this genus*: Barnesiella viscericola*^T^ (91.9%) and *Barnesiella intestinihominis* (91.8%). POCP analysis confirmed the placement of strain E21 within the *Barnesiella* with values of 59.3% and 59.8% to *B. viscericola*^T^ (91.9%) and *B. intestinihominis*, respectively. ANI values to all close relatives were below 95.0%, suggesting this isolate represents a novel species. The assignment as a novel species within *Barnesiella* was supported by GTDB-Tk assignment to ‘Barnesiella sp003150885’. Within the genome, 221 CAZymes were identified along with the utilisation of cellulose and starch. KEGG-based analysis identified the presence of the following pathways: acetate production from acetyl-CoA (EC:2.3.1.8, 2.7.2.1), propionate production from propanoyl-CoA (EC:2.3.1.8, 2.7.2.1), l-glutamate production from ammonia via l-glutamine (EC:6.3.1.2, 1.4.1.-), folate (vitamin B9) biosynthesis from 7,8-dihydrofolate (EC:1.5.1.3) and riboflavin (vitamin B2) biosynthesis from GTP (EC:3.5.4.25, 3.5.4.26, 1.1.1.193, 3.1.3.104, 4.1.99.12, 2.5.1.78, 2.5.1.9, 2.7.1.26, 2.7.7.2). The type strain, E21^T^ (=DSM 107469^T^), was isolated from human faeces. The G + C content of genomic DNA is 40.3%.

#### Description of *Blautia ammoniilytica* sp. nov

*B. ammoniilytica* (N.L. neut. n. *ammonium*, ammonia; N.L. fem. adj. *lytica*, able to loose, able to dissolve; from Gr. fem. adj. *lytike*, able to loose, dissolving; N.L. fem. adj. *ammoniilytica*, ammonia-degrading, to reflect the activity of the bacterium). The species was identified as a member of the genus *Blautia* based on a 16S rRNA gene sequence similarity of 95.5% to *Blautia faecis*. However, similarity to the type species, *Blautia coccoides*, was only 92.6%. The assignment of the isolate to *Blautia* was supported by POCP values of 57 and 58.1% to *Blautia wexlerae* and *Blautia obeum*, respectively, whilst a value of 39.2% to *B. coccoides* suggests genus-level differentiation. ANI values against all members of the genus *Blautia* were below 95%. Both 16S rRNA gene sequence similarity and POCP suggest that *B. ammoniilytica* does not belong to the novel genus ‘*Hoministercoradaptatus’* also described in this study (see below), with values of 93.0 and 50.3%, respectively, to strain Sanger_23. In addition, phylogenomic placement confirmed this isolate resides between *Blautia* species with validly published names, adding to this monophyletic group. As such strain Sanger_23 is proposed as a novel species within the existing genus *Blautia*. GTDB assignment identified strain Sanger_23 as ‘Blautia_A sp900066505’. Whilst 196 CAZymes were identified within the genome, only starch was suggested as a carbon source. KEGG analysis identified a total of 117 transporters, 4 secretion genes, and 591 enzymes within the genome. This included pathways for acetate production from acetyl-CoA (EC:2.3.1.8, 2.7.2.1), propionate production from propanoyl-CoA (EC:2.3.1.8, 2.7.2.1), sulfide and l-serine utilisation to produce l-cysteine and acetate (EC:2.3.1.30, 2.5.1.47), l-glutamate production from ammonia via l-glutamine (EC:6.3.1.2, 1.4.1.-), as well as folate (vitamin B9) biosynthesis from 7,8-dihydrofolate (EC:1.5.1.3). The type strain, Sanger_23^T^ (=DSM 102163^T^), was isolated from human faeces. Its G + C content of genomic DNA is 43.1%.

#### Description of *Blautia acetigignens* sp. nov

*Blautia acetigignens* (L. neut. n. *acetum*, vinegar, used to refer to acetic acid; L. v. *gignere*, to produce; N.L. part. adj. *acetigignens*, vinegar- or acetic acid-producing). The species was identified as a member of the genus *Blautia*, based on 16S rRNA similarity of 98.0% to *B. faecis*, 96.1% to *B. obeum* and 94.6% to the type species, *B. coccoides*. While the POCP to *B. coccoides* was below 50%, the value to *B. obeum* was 60.9%, supporting their placement within the same genus. The placement within *Blautia* is further supported by the isolates GTDB-Tk identification as ‘Blautia_A sp900066145’. ANI values to all close relatives and to ‘*Blautia ammoniilytica’* described in this study (see above) were below 95%. Within the genome 262 CAZymes were identified along with the predicted use of starch. KEGG-based analysis suggested the presence of the following pathways: acetate production from acetyl-CoA (EC:2.3.1.8, 2.7.2.1), propionate production from propanoyl-CoA (EC:2.3.1.8, 2.7.2.1), l-glutamate production from ammonia via l-glutamine (EC:6.3.1.2, 1.4.1.-), cobalamin (vitamin B12) biosynthesis from cobinamide (EC:2.5.1.17, 6.3.5.10, 6.2.1.10, 2.7.1.156), folate (vitamin B9) biosynthesis from 7,8-dihydrofolate (EC:1.5.1.3) and riboflavin (vitamin B2) biosynthesis from GTP (EC:3.5.4.25, 3.5.4.26, 1.1.1.193, 3.1.3.104, 4.1.99.12, 2.5.1.78, 2.5.1.9, 2.7.1.26, 2.7.7.2). The type strain, Sanger_28^T^ (=DSM 102165^T^), was isolated from human faeces. The G + C content of genomic DNA is 44.4%.

#### Description of *Bovifimicola* gen. nov

*Bovifimicola* (L. masc./fem. n. *bos*, an ox, a bull, a cow; L. neut. n. *fimum*, dung; N.L. suffix masc./fem. *cola*, an inhabitant of; N.L. fem. n. *Bovifimicola*, a microbe frequently occurring in the faeces of cattle). This taxon is named referring to the microbial ecosystem with combined prevalence and mean relative abundance for this taxon, although originally isolated from human faeces. Based on 16S rRNA gene sequence similarity, the closest relatives are members of the genera *Hespellia* (*Hespellia porcina*, 93.7%; *Hespellia stercorisuis*^T^, 93.3%) and *Ruminococcus* (*Ruminococcus gnavus*, 93.7%). POCP analysis confirmed that strain Sanger_97 represents a distinct genus to both *Hespellia* (*H. stercorisuis*^T^, 36.3%) and *Ruminococcus* (*R. gnavus*, 36.4%) as all POCP values to close relatives were below 50%. GTDB-Tk supported the creation of a novel genus, placing strain Sanger_97 within the predicted genus ‘CAG-603’. The type species of this genus is *Bovifimicola ammoniilytica*.

#### Description of *Bovifimicola ammoniilytica* sp. nov

*B. ammoniilytica* (N.L. neut. n. *ammonium*, ammonia; N.L. fem. adj. *lytica*, able to loose, able to dissolve; from Gr. fem. adj. *lytike*, able to loose, dissolving; N.L. fem. adj. *ammoniilytica*, ammonia-degrading, to reflect the activity of the bacterium). KEGG analysis identified pathways for acetate production from acetyl-CoA (EC:2.3.1.8, 2.7.2.1), propionate production from propanoyl-CoA (EC:2.3.1.8, 2.7.2.1), l-glutamate production from ammonia via l-glutamine (EC:6.3.1.2, 1.4.1.-), folate (vitamin B9) biosynthesis from 7,8-dihydrofolate (EC:1.5.1.3) and riboflavin (vitamin B2) biosynthesis from GTP (EC:3.5.4.25, 3.5.4.26, 1.1.1.193, 3.1.3.104, 4.1.99.12, 2.5.1.78, 2.5.1.9, 2.7.1.26, 2.7.7.2). This species was commonly identified within wastewater microbiome (14.1% of samples, mean relative abundance of 0.01%), bovine gut (6.3% of samples, mean relative abundance of 0.09%) and human gut (2.6% of samples, mean relative abundance of 0.08%) samples. The type strain, Sanger_97^T^ (=DSM 102166^T^ = CCUG 68796^T^), was isolated from human faeces. The G + C content of genomic DNA is 34.0%.

#### Description of *Brotolimicola* gen. nov

*Brotolimicola* (Gr. masc. n. *brotos*, a mortal human; L. masc. n. *limus*, dung; N.L. suffix masc./fem. *cola*, an inhabitant of; *Brotolimicola*, a microbe from the faeces of humans). The closest relatives, based on 16S rRNA gene sequence similarity, are *Kineothrix alysoides*^T^ (93.1%) and *L. multipara*^T^ (92.2%). POCP analysis confirmed the species represents a distinct genus to *K. alysoides*^T^ (≤41.5%) and *L. multipara*^T^ (≤34.0%). The type species of this genus is *Brotolimicola acetigignens*.

#### Description of *Brotolimicola acetigignens* sp. nov

*B. acetigignens* (L. neut. n. *acetum*, vinegar, used to refer to acetic acid; L. v. *gignere*, to produce; N.L. part. adj. *acetigignens*, vinegar- or acetic acid-producing). This species contains at least 278 CAZymes and are predicted to utilise arbutin, salicin, cellobiose, starch and cellulose. KEGG analysis identified pathways for acetate production identified from acetyl-CoA (EC:2.3.1.8, 2.7.2.1), propionate production from propanoyl-CoA (EC:2.3.1.8, 2.7.2.1), sulfide and l-serine utilised to produce l-cysteine and acetate (EC:2.3.1.30, 2.5.1.47), l-glutamate production from ammonia via l-glutamine (EC:6.3.1.2, 1.4.1.-), cobalamin (vitamin B12) biosynthesis from cobinamide (EC:2.5.1.17, 6.3.5.10, 6.2.1.10, 2.7.1.156) and folate (vitamin B9) biosynthesis from 7,8-dihydrofolate (EC:1.5.1.3). The type strain, f_CXY^T^ (=DSM 107528^T^), was isolated from human faeces. The G + C content of genomic DNA was 46.0% for both isolates. The placement and description of this species are based on two isolates, f_CXY^T^ and f_CSY (=DSM 107475). Only features that were consistent between these two isolates are described above as those of the species.

#### Description of *Brotomerdimonas* gen. nov

*Brotomerdimonas* (Gr. masc. n. *brotos*, a mortal human; L. fem. n. *merda*, dung; L. fem. n. *monas*, a monad; N.L. fem. n. *Brotomerdimonas*, a microbe from the faeces of humans). Based on 16S rRNA gene sequence similarity, the closest relatives are members of *Anaerovorax* (*Anaerovorax odorimutans*^T^, 90.9%) and *Eubacterium* (*Eubacterium infirmum*, 90.8%). POCP analysis confirmed that strain Sanger_12 represents a distinct genus to both *Anaerovorax* (*A. odorimutans*^T^, 41.2%) and *Eubacterium* (*E. infirmum*, 37.0%) as all POCP values to close relatives were below 50%. GTDB-Tk supported the creation of a novel genus, placing strain Sanger_12 within the predicted genus ‘UBA1191’. The type species of this genus is *Brotomerdimonas butyrica*.

#### Description of *Brotomerdimonas butyrica* sp. nov

*B. butyrica* (Gr. neut. n. *boutyron* (Latin transliteration *butyrum*); Gr. fem. adj. suff. -*ica*, suffix used with the sense of belonging to; N.L. fem. adj. *butyrica*, related to butter, butyric). KEGG analysis identified pathways for acetate production from acetyl-CoA (EC:2.3.1.8, 2.7.2.1), butyrate production from butanoyl-CoA (EC:2.8.3.8), propionate production from propanoyl-CoA (EC:2.3.1.8, 2.7.2.1), folate (vitamin B9) biosynthesis from 7,8-dihydrofolate (EC:1.5.1.3) and riboflavin (vitamin B2) biosynthesis from GTP (EC:3.5.4.25, 3.5.4.26, 1.1.1.193, 3.1.3.104, 4.1.99.12, 2.5.1.78, 2.5.1.9, 2.7.1.26, 2.7.7.2). This species was identified to be a common member of both the pig gut (54.5%) and human gut (40.4%), although sub-dominant at <0.1% mean relative abundance respectively. The type strain, Sanger_12^T^ (=DSM 102176^T^ = LMG 29485^T^), was isolated from human faeces. The G + C content of genomic DNA is 47.8%.

#### Description of *Brotonthovivens* gen. nov

*Brotonthovivens* (Gr. masc. n. *brotos*, a mortal human; Gr. masc. n. *onthos*, dung; L. pres. part. *vivens*, living; N.L. fem. n. *Brotonthovivens*, a microbe from the faeces of humans). Based on 16S rRNA gene sequence similarity, the closest relatives are members of *Roseburia*: *Roseburia inulinivorans* (94.6%), *Roseburia intestinalis* (93.6%) and *Roseburia hominis* (93.5%). As no 16S rRNA gene sequence or genome exist for the type species of the genus, *Roseburia cecicola*^T^, no comparisons could be conducted. POCP analysis confirmed that strain Sanger_109 represents a distinct genus to *Roseburia* as all POCP values to close relatives were below 50%. GTDB-Tk supported the placement of strain Sanger_109 within the genus ‘Eubacterium_I’ and a representative of ‘Eubacterium_I sp900066595’. The type species of this genus is *Brotonthovivens ammoniilytica*.

#### Description of *Brotonthovivens ammoniilytica* sp. nov

*B. ammoniilytica* (N.L. neut. n. *ammonium*, ammonia; N.L. fem. adj. *lytica*, able to loose, able to dissolve; from Gr. fem. adj. *lytike*, able to loose, dissolving; N.L. fem. adj. *ammoniilytica*, ammonia-degrading, to reflect the activity of the bacterium). Within the genome, 138 CAZymes were identified along with predicted utilisation of starch. KEGG analysis identified pathways for acetate production from acetyl-CoA (EC:2.3.1.8, 2.7.2.1), butyrate production from butanoyl-CoA (EC:2.8.3.8), propionate production from propanoyl-CoA (EC:2.3.1.8, 2.7.2.1), l-glutamate production from ammonia via l-glutamine (EC:6.3.1.2, 1.4.1.-), cobalamin (vitamin B12) biosynthesis from cobinamide (EC:2.5.1.17, 6.3.5.10, 6.2.1.10, 2.7.1.156), folate (vitamin B9) biosynthesis from 7,8-dihydrofolate (EC:1.5.1.3), riboflavin (vitamin B2) biosynthesis from GTP (EC:3.5.4.25, 3.5.4.26, 1.1.1.193, 3.1.3.104, 4.1.99.12, 2.5.1.78, 2.5.1.9, 2.7.1.26, 2.7.7.2). The type strain, Sanger_109^T^ (=DSM 102148^T^), was isolated from human faeces. The G + C content of genomic DNA is 42.4%.

#### Description of *Clostridium ammoniilyticum* sp. nov

*Clostridium ammoniilyticum* (N.L. neut. n. *ammonium*, ammonia; N.L. neut. adj. *lyticum*, able to loose, able to dissolve; from Gr. neut. adj. *lytikon*, able to loose, dissolving; N.L. neut. adj. *ammoniilyticum*, ammonia-degrading, to reflect the activity of the bacterium). The species was identified as a member of the genus *Clostridium*, based on POCP values of >50% to multiple existing species within this genus: *Clostridium cocleatum* (50.2%), *Clostridium saccharogumia* (52.9%), and *Clostridium spiroforme* (53.5%). ANI values to all close relatives were below 95% and 16S rRNA gene sequence similarity values below 94%. Comparison against *Clostridium butyricum*, the type species of *Clostridium*, was not conducted as it was not within the 50 most similar species, hence ignored by Protologger. This further highlights the need for the reclassification of *Clostridium* into multiple genera. Isolates of this species contained an average of 170 CAZymes and the predicted utilisation of starch, arbutin, salicin, cellobiose and glucose as carbon sources. KEGG analysis predicted the presence of pathways for propionate production from propanoyl-CoA (EC:2.3.1.8, 2.7.2.1), l-glutamate production from ammonia was identified via l-glutamine (EC:6.3.1.2, 1.4.1.-) and folate (vitamin B9) biosynthesis from 7,8-dihydrofolate (EC:1.5.1.3). The type strain, H4_15^T^ (=DSM 108253^T^), was isolated from human faeces. Taxonomic placement and description of this species is based on three isolates as both the additional isolates (H6_14 (=DSM 108252) and H3_29 (=DSM 108213)) had an ANI values >95% to the type strain. Only features that were consistent between these three isolates are described above as those of the species. The G + C content of genomic DNA is between 29.2 and 29.5%.

#### Description of *Coprococcus ammoniilyticus* sp. nov

*Coprococcus ammoniilyticus* (N.L. neut. n. *ammonium*, ammonia; N.L. masc. adj. *lyticus*, able to loose, able to dissolve; from Gr. fem. adj. *lytikos*, able to loose, dissolving; N.L. masc. adj. *ammoniilyticus*, ammonia-degrading, to reflect the activity of the bacterium). The species was identified as a member of the genus *Coprococcus* based on 16S rRNA gene sequence similarity and POCP values of 96.5% and 62.3% to the type species *C. eutactus*, respectively. ANI values to all close relatives were below 95%, suggesting this isolate as a novel species, which was supported by GTDB-Tk assignment to ‘Coprococcus sp900066115’. An ANI value of 80.4% to ‘*Coprococcus aceti’* confirms that these isolates represent two novel species within the genus *Coprococcus*. Within the genome, 120 CAZymes were identified along with the utilisation of glucose, cellulose and starch. KEGG-based analysis identified the presence of the following pathways: acetate production from acetyl-CoA (EC:2.3.1.8, 2.7.2.1), propionate production from propanoyl-CoA (EC:2.3.1.8, 2.7.2.1), l-glutamate production from ammonia via l-glutamine (EC:6.3.1.2, 1.4.1.-) and riboflavin (vitamin B2) biosynthesis from GTP (EC:3.5.4.25, 3.5.4.26, 1.1.1.193, 3.1.3.104, 4.1.99.12, 2.5.1.78, 2.5.1.9, 2.7.1.26, 2.7.7.2). The type strain, H1_22^T^ (=DSM 108312^T^), was isolated from human faeces. The G + C content of genomic DNA is 41.0%.

#### Description of *Coprococcus aceti* sp. nov

*C. aceti* (L. neut. n. *acetum*, vinegar; L. gen. neut. n. *aceti*, of vinegar). The species was identified as a member of the genus *Coprococcus*. The comparison of 16S rRNA gene sequences identified the highest match to the type species of *Coprococcus*, *Coprococcus eutactus* (99.4%). GTDB-Tk supported the placement of strain H2_11 within the *Coprococcus*, placed as ‘Coprococcus eutactus_A’. However, dDDH comparison to the type strains genome via TYGS^35^ confirmed that strain H2_11 belongs to a species distinct from *C. eutactus*. ANI values to all close relatives were below 95%, confirming this isolate represents a novel species and 80.4% to ‘*C. ammoniilyticus’* confirms that these isolates represent two novel species within the genus *Coprococcus*. Within the genome, 162 CAZymes were identified along with the utilisation of starch and cellulose. KEGG-based analysis identified the presence of the following pathways: acetate production identified from acetyl-CoA (EC:2.3.1.8, 2.7.2.1), butyrate production from butanoyl-CoA (EC:2.8.3.8), propionate production from propanoyl-CoA (EC:2.3.1.8, 2.7.2.1), l-glutamate production from ammonia via l-glutamine (EC:6.3.1.2, 1.4.1.-), folate (vitamin B9) biosynthesis from 7,8-dihydrofolate (EC:1.5.1.3) and riboflavin (vitamin B2) biosynthesis from GTP (EC:3.5.4.25, 3.5.4.26, 1.1.1.193, 3.1.3.104, 4.1.99.12, 2.5.1.78, 2.5.1.9, 2.7.1.26, 2.7.7.2). The type strain, H2_11^T^ (=DSM 107541^T^ = JCM 31265^T^), was isolated from human faeces. The G + C content of genomic DNA is 43.7%.

#### Description of *Dorea acetigenes* sp. nov

*Dorea acetigenes* (L. neut. n. *acetum*, vinegar; Gr. v. *gennaô*, to produce; N.L. part. adj. *acetigenes*, producing acetate). The species was identified as a member of the genus *Dorea*, based on a POCP value to the type species of the genus, *D. formicigenerans*^T^, of 52.3%. GTDB-Tk supported placement of Sanger_03 within the genus *Dorea*. Strikingly, 16S rRNA gene sequence similarity to *Dorea formicigenerans*^T^ was only 92.7%. Higher similarity values were obtained to *Faecalimonas* (*Faecalimonas umbilicata*^T^, 95.5%) and *Faecalicatena* (*Faecalicatena contorta*^T^, 94.8%). POCP comparison was highest against *D. formicigenerans*^T^ (52.3%), while only 42.1% to *Faecalicatena contorta*^T^. Separation from the species ‘*Dorea amylophila’* and ‘*Dorea ammoniilytica’* was confirmed via an ANI values of 85.7% and 82.2%, respectively, between the genomes of the type strains. Whilst 224 CAZymes were identified within the genome, only starch was suggested as a carbon source. KEGG analysis identified a total of 91 transporters, 8 secretion genes and 512 enzymes were identified. This included pathways for acetate production from acetyl-CoA (EC:2.3.1.8, 2.7.2.1), propionate production from propanoyl-CoA (EC:2.3.1.8, 2.7.2.1), sulfide and l-serine utilised to produce l-cysteine and acetate (EC:2.3.1.30, 2.5.1.47), l-glutamate production from ammonia via l-glutamine (EC:6.3.1.2, 1.4.1.-), as well as folate (vitamin B9) biosynthesis from 7,8-dihydrofolate (EC:1.5.1.3). The type strain, Sanger_03^T^ (=DSM 102260^T^), was isolated from human faeces. Its G + C content of genomic DNA is 43.6%.

#### Description of *Dorea ammoniilytica* sp. nov

*D. ammoniilytica* (N.L. neut. n. *ammonium*, ammonia; N.L. fem. adj. *lytica*, able to loose, able to dissolve; from Gr. fem. adj. *lytike*, able to loose, dissolving; N.L. fem. adj. *ammoniilytica*, ammonia-degrading, to reflect the activity of the bacterium). The species was identified as a member of the genus *Dorea*, based on a POCP value of 56.2% to the type species of the genus, *D. formicigenerans*^T^, and of 58.9% to *D. longicatena*. GTDB-Tk supported placement of the type strain, Sanger_02, as a member of the *Dorea*. Interestingly, 16S rRNA gene sequence similarity to *D. formicigenerans*^T^ was only 92.7%. Higher similarity values were obtained to members of the *Faecalicatena* genus: *F. contorta*^T^ (96.6%) and *F. fissicatena* (96.1%). However, POCP values to these species’ genomes were below 50% and above 50% to both *D. formicigenerans*^T^ (56.2%) and *D. longicatena* (58.9%). These values confirm that strain Sanger_02 is a novel species of *Dorea*. Separation from the species ‘*D. acetigenes’* and ‘*D. amylophila’* was confirmed via an ANI values of 82.2% and 80.6%, respectively, between the genomes of the type strains. The genome contained 125 CAZymes, facilitating the predicted utilisation of both starch and glucose as carbon sources. KEGG analysis identified a total of 93 transporters, 7 secretion genes and 513 enzymes within the genome. This included pathways for acetate production from acetyl-CoA (EC:2.3.1.8, 2.7.2.1), butyrate production from butanoyl-CoA (EC:2.8.3.8), propionate production from propanoyl-CoA (EC:2.3.1.8, 2.7.2.1), sulfide and l-serine utilised to produce l-cysteine and acetate (EC:2.3.1.30, 2.5.1.47), l-glutamate production from ammonia via l-glutamine (EC:6.3.1.2, 1.4.1.-), cobalamin (vitamin B12) biosynthesis from cobinamide (EC:2.5.1.17, 6.3.5.10, 6.2.1.10, 2.7.1.156), as well as folate (vitamin B9) biosynthesis from 7,8-dihydrofolate (EC:1.5.1.3). The type strain, Sanger_02^T^ (=DSM 102136^T^), was isolated from human faeces. Its G + C content of genomic DNA is 43.3%.

#### Description of *Dorea amylophila* sp. nov

*D. amylophila* (Gr. neut. n. *amylon*, starch; N.L. fem. adj. *phila* (from Gr. fem. adj. *phile*) loving; N.L. fem. adj. *amylophila*, starch-loving). The species was identified as a member of the genus *Dorea*, based on a 16S rRNA gene sequence similarity of 100.0% to *Dorea longicatena* and 95.4% to the type species of this genus, *D. formicigenerans*^T^. This is supported by POCP values of 74.8% and 62.3%, respectively. While the 16S rRNA gene sequence similarity suggests this isolate is a strain of *D. longicatena*, the ANI value between these genomes was 91.2%, suggesting it as a novel species. GTDB-Tk identified the isolate as ‘Dorea longicatena_B’. This is due to the need to split *D. longicatena* into two distinct species based on genomic comparison. Separation from the species ‘*D. acetigenes’* and ‘*D. ammoniilytica’* was confirmed via an ANI values of 85.7% and 80.6%, respectively, between the genomes of the type strains. Within the genome, 160 CAZymes were identified along with the predicted utilisation of glucose, arbutin, salicin, trehalose and starch as carbon sources. KEGG analysis identified a total of 97 transporters, 10 secretion genes and 507 enzymes. This included pathways for acetate production from acetyl-CoA (EC:2.3.1.8, 2.7.2.1), butyrate production from butanoyl-CoA (EC:2.8.3.8), propionate production from propanoyl-CoA (EC:2.3.1.8, 2.7.2.1), sulfide and l-serine utilised to produce l-cysteine and acetate (EC:2.3.1.30, 2.5.1.47), l-glutamate production from ammonia via l-glutamine (EC:6.3.1.2, 1.4.1.-), folate (vitamin B9) biosynthesis from 7,8-dihydrofolate (EC:1.5.1.3) and riboflavin (vitamin B2) biosynthesis from GTP (EC:3.5.4.25, 3.5.4.26, 1.1.1.193, 3.1.3.104, 4.1.99.12, 2.5.1.78, 2.5.1.9, 2.7.1.26, 2.7.7.2). The type strain, H5_25^T^ (=DSM 108233^T^), was isolated from human faeces. Its G + C content of genomic DNA is 41.2%.

#### Description of *Faecalicatena acetigenes* sp. nov

*Faecalicatena acetigenes* (L. neut. n. *acetum*, vinegar; Gr. v. *gennaô*, to produce; N.L. part. adj. *acetigenes*, producing acetate). The species was identified as a member of the genus, *Faecalicatena*. The comparison of 16S rRNA gene sequences identified matches to members of three genera: *Mediterraneibacter* (*Mediterraneibacter glycyrrhizinilyticus*, 95.3%), *Ruminococcus* (*Ruminococcus torques*, 95.3%) and *Faecalicatena* (*F. orotica*, 94.9%, *F. contorta*^T^, 94.9%, *Faecalicatena fissicatena*, 94.8%). The similarity to these three genera was supported by POCP values above 50% to members of each genus, including; *Mediterraneibacter* (*M. glycyrrhizinilyticus*, 55.8%), *Ruminococcus* (*R. torques*, 57%) and *Faecalicatena* (*F. fissicatena*, 52.2%). GTDB-Tk assigned the isolate to ‘Faecalicatena sp001487105’, an as-yet-uncultured member of this genus. Due to the lack of sequence similarity, either at the 16S rRNA gene sequence or genome level, to the type species of either *Ruminococcus* or *Mediterraneibacter*, the isolate was placed within the *Faecalicatena*. Within the genome, 140 CAZymes were identified along with the utilisation of starch and glucose. KEGG-based analysis identified the presence of the following pathways: acetate production identified from acetyl-CoA (EC:2.3.1.8, 2.7.2.1), propionate production from propanoyl-CoA (EC:2.3.1.8, 2.7.2.1), sulfide and l-serine utilised to produce l-cysteine and acetate (EC:2.3.1.30, 2.5.1.47), l-glutamate production from ammonia via l-glutamine (EC:6.3.1.2, 1.4.1.-), folate (vitamin B9) biosynthesis from 7,8-dihydrofolate (EC:1.5.1.3). The type strain, H2_18^T^ (=DSM 108153^T^), was isolated from human faeces. The G + C content of genomic DNA is 42.4%.

#### Description of *Gallintestinimicrobium* gen. nov

*Gallintestinimicrobium* (L. masc. n. *gallus*, a chicken; L. neut. n. *intestinum*, the gut; N.L. neut. n. *microbium* a microbe; N.L. neut. n. *Gallintestinimicrobium*, a microbe frequently occurring in the intestines of chickens). This taxon is named referring to the microbial ecosystem with combined prevalence and mean relative abundance for this taxon, although originally isolated from human faeces. Based on 16S rRNA gene sequence similarity, the closest relatives are members of *Eisenbergiella* (*Eisenbergiella tayi*, 93.7%) and *Enterocloster* (*Enterocloster bolteae*, 93.3%; *Enterocloster clostridioformis*, 93.1%; *Enterocloster aldenensis*, 93%). POCP analysis confirmed that strain Sanger_16 represents a distinct genus to both *Eisenbergiella* and *Enterocloster* as all POCP values to close relatives were below 50%, including 42.1% to *E. tayi*. The type species of this genus is *Gallintestinimicrobium propionicum*.

#### Description of *Gallintestinimicrobium propionicum* sp. nov

*G. propionicum* (N.L. neut. n. *acidum propionicum*, propionic acid; L. neut. suff. -*icum*, suffix used with the sense of pertaining to; N.L. neut. adj. *propionicum*, pertaining to propionic acid). Within the genome, 289 CAZymes were identified along with the predicted utilisation of arbutin, salicin, trehalose and starch. KEGG analysis identified pathways for acetate production from acetyl-CoA (EC:2.3.1.8, 2.7.2.1), propionate production from propanoyl-CoA (EC:2.3.1.8, 2.7.2.1), sulfide and l-serine utilised to produce l-cysteine and acetate (EC:2.3.1.30, 2.5.1.47), l-glutamate production from ammonia via l-glutamine (EC:6.3.1.2, 1.4.1.-), cobalamin (vitamin B12) biosynthesis from cobinamide (EC:2.5.1.17, 6.3.5.10, 6.2.1.10, 2.7.1.156) and folate (vitamin B9) biosynthesis from 7,8-dihydrofolate (EC:1.5.1.3). This species was most commonly identified within chicken gut samples (50.7%) at 1.48% mean relative abundance. The type strain, Sanger_16^T^ (=DSM 102825^T^), was isolated from human faeces. The G + C content of genomic DNA is 47.4%.

#### Description of *Hominimerdicola* gen. nov

*Hominimerdicola* (L. masc. n. *homo*, a human being; L. fem. n. *merda*, dung; N.L. masc./fem. suff. –*cola*, an inhabitant of; N.L. fem. n. *Hominimerdicola*, a microbe from the faeces of humans). The type isolate was identified to match the previously described, but not validated, species ‘*Ruminococcus bicirculans’* based on 16S rRNA gene sequence similarity (99.7%) and GTDB-Tk placement. While originally placed within the *Ruminococcus*, strain Sanger_31 represents a novel genus based on POCP and GTDB-Tk. POCP analysis confirmed that strain Sanger_31 represents a distinct genus to both *Ruminococcus* (*Ruminococcus flavefaciens*^T^, 34.5%) and all other close relatives as all POCP values were below 50%. GTDB-Tk supported the creation of a novel genus, placing strain Sanger_31 within ‘Ruminococcus_D’, a sub-division of the existing *Ruminococcus* genus. The type species of this genus is *Hominimerdicola aceti*.

#### Description of *Hominimerdicola aceti* sp. nov

*H. aceti* (L. neut. n. *acetum*, vinegar; L. gen. neut. n. *aceti*, of vinegar). The number of CAZymes identified within the type strains genome was 168, facilitating the predicted utilisation of glucose, cellulose and starch. KEGG analysis identified pathways for propionate production from propanoyl-CoA (EC:2.3.1.222, 2.7.2.1), sulfide and l-serine utilised to produce l-cysteine and acetate (EC:2.3.1.30, 2.5.1.47) and l-glutamate production from ammonia via l-glutamine (EC:6.3.1.2, 1.4.1.-). The production of acetate by the 80/3 strain of this species has previously been demonstrated^76^. The type strain, Sanger_31^T^ (=DSM 102216^T^), was isolated from human faeces. The G + C content of genomic DNA is 42.3%.

#### Description of *Hoministercoradaptatus* gen. nov

*Hoministercoradaptatus* (L. masc. n. *homo*, a human being; L. neut. n. *stercus*, dung; L. past part. *adaptatus* adapted to; N.L. masc. n. *Hoministercoradaptatus*, a microbe from the faeces of humans). This isolate was identified as a distinct genus to its closest relatives, *Blautia* spp., based on 16S rRNA gene sequence similarity and POCP values to the type species, *B. coccoides*, of 93.8% and 46.0%, respectively. This was confirmed via GTDB-Tk assigning strain Sanger_32 as a member of ‘Ruminococcus_A’, a genus distinct to that of *Blautia*. The type species of this proposed genus is *Hoministercoradaptatus ammoniilyticus*.

#### Description of *Hoministercoradaptatus ammoniilyticus* sp. nov

*H. ammoniilyticus* (N.L. neut. n. *ammonium*, ammonia; N.L. masc. adj. *lyticus*, able to loose, able to dissolve; from Gr. fem. adj. *lytikos*, able to loose, dissolving; N.L. masc. adj. *ammoniilyticus*, ammonia-degrading, to reflect the activity of the bacterium). The species contains at least 225 CAZymes, however only starch was suggested as a carbon source. KEGG-based analysis identified the presence of pathways for acetate production from acetyl-CoA (EC:2.3.1.8, 2.7.2.1), propionate production from propanoyl-CoA (EC:2.3.1.8, 2.7.2.1), l-glutamate production from ammonia via l-glutamine (EC:6.3.1.2, 1.4.1.-), as well as folate (vitamin B9) biosynthesis from 7,8-dihydrofolate (EC:1.5.1.3). The G + C content of genomic DNA for this species was between 43.7–43.9%. The type strain, Sanger_32^T^ (=DSM 102174^T^), was isolated from human faeces. A second isolate (f_RGN (=DSM 107527)) was also isolated from human faeces with an ANI value of 98.0% to the type strain. Only features that were consistent between these two isolates are described above as those of the species.

#### Description of *Huintestinicola* gen. nov

*Huintestinicola* (Gr. masc./fem. n. *hus*, a pig; L. neut. n. *intestinum*, the gut; N.L. masc./fem. suff. –*cola*, an inhabitant of; N.L. fem. n. *Huintestinicola*, a microbe frequently occurring in the intestines of pigs). This taxon is named referring to the microbial ecosystem with combined prevalence and mean relative abundance for this taxon, although originally isolated from human faeces. Based on 16S rRNA gene sequence similarity, the closest relatives are members of the genera *Ruminococcus* (*Ruminococcus callidus*, 91.9%; *R. flavefaciens*^T^, 91%) and *Clostridium* (*Clostridium methylpentosum*, 91.1%). POCP analysis confirmed that strain Sanger_06 represents a distinct genus to both *Ruminococcus* (*R. flavefaciens*^T^, 35.5%) and *Clostridium* (*C. methylpentosum*, 33.1%) as all POCP values to close relatives were below 50%. GTDB-Tk supported the creation of a novel genus, placing strain Sanger_06 within the predicted genus ‘CAG-353’. The type species of this genus is *H. butyrica*.

#### Description of *Huintestinicola butyrica* sp. nov

*H. butyrica* (Gr. neut. n. *boutyron* (Latin transliteration *butyrum*); Gr. fem. adj. suff. -*ica*, suffix used with the sense of belonging to; N.L. fem. adj. *butyrica*, related to butter, butyric). The number of CAZymes identified within the type strains genome was 180, facilitating the predicted utilisation of cellulose and starch. KEGG analysis identified pathways for butyrate production from butanoyl-CoA (EC:2.8.3.8), sulfide and l-serine utilised to produce l-cysteine and acetate (EC:2.3.1.30, 2.5.1.47), l-glutamate production from ammonia via l-glutamine (EC:6.3.1.2, 1.4.1.-) and cobalamin (vitamin B12) biosynthesis from cobinamide (EC:2.5.1.17, 6.3.5.10, 6.2.1.10, 2.7.1.156). This species was most commonly identified within pig gut samples (29.2%), although sub-dominant at only 0.1 % mean relative abundance. The type strain, Sanger_06^T^ (=DSM 102115^T^), was isolated from human faeces. The G + C content of genomic DNA is 46.5%.

#### Description of *Laedolimicola* gen. nov

*Laedolimicola* (Gr. masc. n. *laedos*, an unknown bird; L. masc. n. *limus*, dung; N.L. masc./fem. suff. –*cola*, an inhabitant of; N.L. fem. n. *Laedolimicola*, a microbe frequently occurring in the faeces of birds). This taxon is named referring to the microbial ecosystem with combined prevalence and mean relative abundance for this taxon, although originally isolated from human faeces. Based on 16S rRNA gene sequence similarity, the closest relatives are members of the genera *Faecalicatena* (*Faecalicatena orotica*, 94.3%; *F. fissicatena*, 94.1%; *F. contorta*^T^, 93.9%) and *Eubacterium* (*Eubacterium oxidoreducens*, 94.1%). POCP analysis confirmed that strain Sanger_04 represents a distinct genus to both *Faecalicatena* and *Eubacterium* as all POCP values to close relatives were below 50%. GTDB-Tk supported the creation of a novel genus, placing strain Sanger_04 within the predicted genus ‘GCA-900066575’. The type species of this genus is *L. ammoniilytica*.

#### Description of *Laedolimicola ammoniilytica* sp. nov

*L. ammoniilytica* (N.L. neut. n. *ammonium*, ammonia; N.L. fem. adj. *lytica*, able to loose, able to dissolve; from Gr. fem. adj. *lytike*, able to loose, dissolving; N.L. fem. adj. *ammoniilytica*, ammonia-degrading, to reflect the activity of the bacterium). Within the genome, 186 CAZymes were identified along with the predicted utilisation of glucose, arbutin, salicin, cellobiose, maltose and starch. KEGG analysis identified pathways for propionate production from propanoyl-CoA (EC:2.3.1.222, 2.7.2.1), sulfide and l-serine to produce l-cysteine and acetate (EC:2.3.1.30, 2.5.1.47), l-glutamate production from ammonia via l-glutamine (EC:6.3.1.2, 1.4.1.-), cobalamin (vitamin B12) biosynthesis from cobinamide (EC:2.5.1.17, 6.3.5.10, 6.2.1.10, 2.7.1.156) and folate (vitamin B9) biosynthesis from 7,8-dihydrofolate (EC:1.5.1.3). This species was most commonly identified within chicken gut samples (65.7%), although sub-dominant at 0.35% mean relative abundance. The type strain, Sanger_04^T^ (=DSM 102317^T^), was isolated from human faeces. The G + C content of genomic DNA is 49.8%.

#### Description of *Megasphaera butyrica* sp. nov

*Megasphaera butyrica* (Gr. neut. n. *boutyron* (Latin transliteration *butyrum*); Gr. fem. adj. suff. -*ica*, suffix used with the sense of belonging to; N.L. fem. adj. *butyrica*, related to butter, butyric). The species was identified as a member of the genus *Megasphaera* based on POCP values >50% to five existing species belonging to this genus, including the type species *Megasphaera elsdenii* (69.5%). ANI values to all close relatives were below 95%, suggesting this isolate as a novel species. This was confirmed by GTDB-Tk identification as ‘Megasphaera sp900066485’. Within the genome, 126 CAZymes were identified along with the utilisation of starch, glucose and sucrose. KEGG-based analysis identified the presence of the following pathways: butyrate production from butanoyl-CoA (EC:2.8.3.8), sulfide and l-serine utilised to produce l-cysteine and acetate (EC:2.3.1.30, 2.5.1.47) and cobalamin (vitamin B12) biosynthesis from cobinamide (EC:2.5.1.17, 6.3.5.10, 6.2.1.10, 2.7.1.156). The type strain, Sanger_24^T^ ( = DSM 102144^T^), was isolated from human faeces. The G + C content of genomic DNA is 52.6%.

#### Description of *Muricoprocola* gen. nov

*Muricoprocola* (L. masc. n. *mus*, a mouse; Gr. fem. n. *kopros*, dung; N. L. masc./fem. suff. -*cola*, an inhabitant of; N.L. fem. n. *Muricoprocola*, a microbe frequently occurring in the faeces of mice). This taxon is named referring to the microbial ecosystem with combined prevalence and mean relative abundance for this taxon, although originally isolated from human faeces. Based on 16S rRNA gene sequence similarity, the closest relatives are members of the genus *Lacrimispora* (*Lacrimispora saccharolytica*, 93.9%; *L. amygdalina*, 93.7%; *Lacrimispora indolis*, 93.3%; *L. sphenoides*, 93.3%). POCP analysis confirmed that strain Sanger_29 represents a distinct genus as all POCP values to close relatives were below 50%. The type species of this genus is *Muricoprocola aceti*.

#### Description of *Muricoprocola aceti* sp. nov

*M. aceti* (L. neut. n. *acetum*, vinegar; L. gen. neut. n. *aceti*, of vinegar). Within the genome, 158 CAZymes were identified along with the predicted utilisation of cellulose and starch. KEGG analysis identified pathways for acetate production from acetyl-CoA (EC:2.3.1.8, 2.7.2.1), propionate production from propanoyl-CoA (EC:2.3.1.8, 2.7.2.1), l-glutamate production from ammonia via l-glutamine (EC:6.3.1.2, 1.4.1.-), cobalamin (vitamin B12) biosynthesis from cobinamide (EC:2.5.1.17, 6.3.5.10, 6.2.1.10, 2.7.1.156) and folate (vitamin B9) biosynthesis from 7,8-dihydrofolate (EC:1.5.1.3). This species was most commonly identified within mouse gut samples (7.9%) at 1.95% mean relative abundance. The type strain, Sanger_29^T^ (=DSM 102151^T^), was isolated from human faeces. The G + C content of genomic DNA is 43.0%.

#### Description of *Muriventricola* gen. nov

*Muriventricola* (L. masc. n. *mus*, a mouse; L. masc. n. *venter*, the belly; N.L. masc./fem. suff. –*cola*, an inhabitant of; N.L. fem. n. *Muriventricola*, a microbe frequently occurring in the intestines of mice). This taxon is named referring to the microbial ecosystem with combined prevalence and mean relative abundance for this taxon, although originally isolated from human faeces. The closest relatives, based on 16S rRNA gene sequence similarity, are *Pseudoflavonifractor capillosus* (95.2%) and *Flavonifractor plautii* (94.9%). POCP analysis confirmed the species as representing a distinct genus to *P. capillosus* (<46.5%) and *F. plautii* (<46.3%). This is supported by the genome tree which shows clear separation from all close relatives. The type species of this genus is *Muriventricola aceti*. The type species is *Muriventricola aceti*.

#### Description of *Muriventricola aceti* sp. nov

*M. aceti* (L. neut. n. *acetum*, vinegar; L. gen. neut. n. *aceti*, of vinegar). The number of CAZymes identified within the genome of strains of this species ranged from 105–116. Isolates were predicted to utilise starch. KEGG analysis identified pathways for acetate production from acetyl-CoA (EC:2.3.1.8, 2.7.2.1), propionate production from propanoyl-CoA (EC:2.3.1.8, 2.7.2.1), sulfide and l-serine utilised to produce l-cysteine and acetate (EC:2.3.1.30, 2.5.1.47) as well as folate (vitamin B9) biosynthesis from 7,8-dihydrofolate (EC:1.5.1.3). This species was most commonly identified within mouse gut samples (67.7%) at 1.0% mean relative abundance. The type strain, H5_61^T^ (=DSM 108267^T^), was isolated from human faeces. The G + C content of genomic DNA ranges from 55.8 – 56.3%. This placement and description of this species is based on two isolates, H5_61^T^ and H4_62 (=DSM 108264). Only features that were consistent between these two isolates are described above as those of the species.

#### Description of *Oscillibacter acetigenes* sp. nov

*O. acetigenes* (L. neut. n. *acetum*, vinegar; Gr. v. *gennaô*, to produce; N.L. part. adj. *acetigenes*, producing acetate). The species was identified as a member of the genus *Oscillibacter*, based on 16S rRNA gene sequence similarity of 95.5% to both existing members of the genus, *Oscillibacter ruminantium* and *Oscillibacter valericigenes*^T^. This was supported by POCP values of 52.3 and 47.0% to each member, respectively. ANI values to both members were below 95%, confirmed by GTDB-Tk identification as ‘Oscillibacter-sp900066435’. This species contains an average of 111 CAZymes. Starch was uniquely predicted as a carbon source. Genomic prediction identified the following pathways: acetate production from acetyl-CoA (EC:2.3.1.8, 2.7.2.1), propionate production from propanoyl-CoA (EC:2.3.1.8, 2.7.2.1), sulfide and l-serine utilised to produce l-cysteine and acetate (EC:2.3.1.30, 2.5.1.47). The type strain, H4_59^T^ (=DSM 108348^T^), was isolated from human faeces. A second isolate (H2_39 (=DSM 108347)) was also isolated from human faeces with an ANI value of 99.0% to the type strain. The G + C content of genomic DNA is between 59.2–59.9%. Only features that were consistent between these two isolates are described above as those of the species.

#### Description of *Phocaeicola fibrisolvens* sp. nov

*P. fibrisolvens* (L. fem. n. *fibra*, a fibre, filament; L. pres. part. *solvens*, dissolving; N.L. part. adj. *fibrisolvens*, fibre-dissolving). The species was identified as a member of the newly proposed genus *Phocaeicola*. The comparison of 16S rRNA gene sequences identified the highest matches to existing members of this genus, including *Phocaeicola coprocola* (94.6%), and low similarity to the type species of the next closest genus *Bacteroides* (*B. fragilis*^T^, 89.2%). POCP analysis also confirmed the placement of strain Sanger_21 within the *Phocaeicola* with values >50% to multiple members, the highest being 73.0% to *Phocaeicola plebeius*. ANI values to all close relatives were below 95%. Within the genome, 372 CAZymes were identified along with the utilisation of starch. KEGG-based analysis identified the presence of the following pathways: acetate production from acetyl-CoA (EC:2.3.1.8, 2.7.2.1), propionate production from propanoyl-CoA (EC:2.3.1.8, 2.7.2.1), l-glutamate production from ammonia via l-glutamine (EC:6.3.1.2, 1.4.1.-), folate (vitamin B9) biosynthesis from 7,8-dihydrofolate (EC:1.5.1.3) and riboflavin (vitamin B2) biosynthesis from GTP (EC:3.5.4.25, 3.5.4.26, 1.1.1.193, 3.1.3.104, 4.1.99.12, 2.5.1.78, 2.5.1.9, 2.7.1.26, 2.7.7.2). The type strain, Sanger_21^T^ (=DSM 102146^T^), was isolated from human faeces. The G + C content of genomic DNA is 46.4%.

#### Description of *Porcipelethomonas* gen. nov

*P.* (L. masc. n. *porcus*, a piglet; Gr. masc. n. *pelethos*, dung; L. fem. n. *monas*, a monad; N.L. fem. n. *Porcipelethomonas*, a microbe frequently occurring in the faeces of pigs). This taxon is named referring to the microbial ecosystem with combined prevalence and mean relative abundance for this taxon, although originally isolated from human faeces. Based on 16S rRNA gene sequence similarity, the closest relatives are *Ruminococcus* (*R. flavefaciens*^T^, 93.1%; *Ruminococcus champanellensis*, 92.9%; *R. callidus*, 92.2%). POCP analysis confirmed that strain Sanger_90 represents a distinct genus to *Ruminococcus* (*R. flavefaciens*^T^, 40.3%; *R. champanellensis*, 46.7%; *R. callidus*, 45.4%) as all POCP values to close relatives were below 50%. GTDB-Tk supported the creation of a novel genus, placing strain Sanger_90 within the predicted genus ‘UBA1394’. The type species of this genus is *Porcipelethomonas ammoniilytica*.

#### Description of *Porcipelethomonas ammoniilytica* sp. nov

*P. ammoniilytica* (N.L. neut. n. *ammonium*, ammonia; N.L. fem. adj. *lytica*, able to loose, able to dissolve; from Gr. fem. adj. *lytike*, able to loose, dissolving; N.L. fem. adj. *ammoniilytica*, ammonia-degrading, to reflect the activity of the bacterium). KEGG analysis identified pathways for propionate production from propanoyl-CoA (EC:2.3.1.222, 2.7.2.1), sulfide and l-serine utilised to produce l-cysteine and acetate (EC:2.3.1.30, 2.5.1.47) and l-glutamate production from ammonia via l-glutamine (EC:6.3.1.2, 1.4.1.-). This species was most commonly identified within pig gut (26.3%) and human gut (7.4%) samples, although sub-dominant at only 0.1% and 0.2% mean relative abundance respectively. The type strain, Sanger_90^T^ (=DSM 102167^T^), was isolated from human faeces. The G + C content of genomic DNA is 36.9%.

#### Description of *Roseburia amylophila* sp. nov

*R. amylophila* (Gr. neut. n. *amylon*, starch; N.L. fem. adj. *phila* (from Gr. fem. adj. *phile*) loving; N.L. fem. adj. *amylophila*, starch-loving). The species was identified as a member of the genus *Roseburia* based on POCP values >50% to four existing species belonging to this genus; however, no genome exists for the type species *R. cecicola*. ANI values to all close relatives were below 95%, suggesting this isolate as a novel species. Inconsistency in taxonomic assignment was observed between methods as the highest match based on 16S rRNA gene sequence similarity was *E. oxidoreducens* (95.5%) and GTDB assignment was to the novel genus ‘CAG-45’. However, after *E. oxidoreducens*, the next best matches based on 16S rRNA gene similarity were to *Roseburia faecis* (95.3%), *R. hominis* (95.1%) and *R. intestinalis* (95.1%). Placement within the genome tree also showed strain Sanger_19 to be monophyletic with the existing *Roseburia* species. Based on the genome tree, we observed that *Eubacterium* is a taxonomically incongruent genus and requires reclassification. Within the genome, 178 CAZymes were identified along with the utilisation of arbutin, salicin, sucrose and starch. KEGG-based analysis identified the presence of the following pathways: propionate production from propanoyl-CoA (EC:2.3.1.8, 2.7.2.1), sulfide and l-serine utilised to produce l-cysteine and acetate (EC:2.3.1.30, 2.5.1.47), l-glutamate production from ammonia was identified via l-glutamine (EC:6.3.1.2, 1.4.1.-), folate (vitamin B9) biosynthesis from 7,8-dihydrofolate (EC:1.5.1.3). The type strain, Sanger_19^T^ (=DSM 102150^T^), was isolated from human faeces. The G + C content of genomic DNA is 40.7%.

#### Description of *Suilimivivens* gen. nov

*Suilimivivens* (L. masc. n. *sus*, a pig; L. masc. n. *limus*, dung; L. pres. part. *vivens* living; N.L. fem. n. *Suilimivivens*, a microbe frequently occurring in the faeces of pigs). This taxon is named referring to the microbial ecosystem with combined prevalence and mean relative abundance for this taxon, although originally isolated from human faeces. Based on 16S rRNA gene sequence similarity, the closest relatives are members of the genera *Kineothrix* (*K. alysoides*^T^, 93.5%) and *Eisenbergiella* (*E.* *tayi*^T^, 92.1%). POCP analysis confirmed that strain Sanger_18 represents a distinct genus to both *Kineothrix* (*K. alysoides*^T^, 44.9%) and *Eisenbergiella* (E. *tayi*^T^, 35.8%) as all POCP values to close relatives were below 50%. GTDB-Tk supported the creation of a novel genus, placing strain Sanger_18 within the predicted genus ‘CAG-95’. The type species of this genus is *Suilimivivens aceti*.

#### Description of *Suilimivivens aceti* sp. nov

*S. aceti* (L. neut. n. *acetum*, vinegar; L. gen. neut. n. *aceti*, of vinegar). The number of CAZymes identified within the type strains genome was 242, facilitating the predicted utilisation of cellulose and starch. KEGG analysis identified pathways for acetate production from acetyl-CoA (EC:2.3.1.8, 2.7.2.1), propionate production from propanoyl-CoA (EC:2.3.1.8, 2.7.2.1), sulfide and l-serine utilised to produce l-cysteine and acetate (EC:2.3.1.30, 2.5.1.47), l-glutamate production from ammonia via l-glutamine (EC:6.3.1.2, 1.4.1.-), folate (vitamin B9) biosynthesis from 7,8-dihydrofolate (EC:1.5.1.3). This species was most commonly identified within pig gut samples (17.3%), although sub-dominant at only 0.5% mean relative abundance. The type strain, Sanger_18^T^ (=DSM 102261^T^), was isolated from human faeces. The G + C content of genomic DNA is 44.4%.

#### Description of *Suonthocola* gen. nov

*Suonthocola* (L. masc. n. *sus*, a pig; Gr. masc. n. *onthos*, dung; N.L. masc./fem. suff. –*cola*, an inhabitant of; N.L. fem. n. *Suonthocola*, a microbe frequently occurring in the faeces of pigs). This taxon is named referring to the microbial ecosystem with combined prevalence and mean relative abundance for this taxon, although originally isolated from human faeces. This isolate was identified as a distinct genus to its closest relatives, *Clostridium nexile* (93.7%) and *Lactonifactor longoviformis* (93.7%), based on 16S rRNA gene sequence similarity. This was supported by POCP values <50% against all close relatives. Further confirmation was obtained by GTDB-Tk assignment as an unnamed genus within the *Lachnospiraceae*. The type species of this genus is *Suonthocola fibrivorans*.

#### Description of *Suonthocola fibrivorans* sp. nov

*Suonthocola fibrivorans* (L. fem. n. *fibra*, fibre; L. v. *vorare*, to devour; N.L. part. adj. *fibrivorans*, fibre-devouring). Within the isolates’ genome, 388 CAZymes were identified along with the predicted use of starch and cellobiose as carbon sources. KEGG analysis identified a total of 132 transporters, 16 secretion genes and 656 enzymes. This included pathways for acetate production from acetyl-CoA (EC:2.3.1.8, 2.7.2.1), propionate production from propanoyl-CoA (EC:2.3.1.8, 2.7.2.1), sulfide and l-serine utilised to produce l-cysteine and acetate (EC:2.3.1.30, 2.5.1.47), l-glutamate production from ammonia via l-glutamine (EC:6.3.1.2, 1.4.1.-), as well as folate (vitamin B9) biosynthesis from 7,8-dihydrofolate (EC:1.5.1.3). This species was most commonly identified within pig gut samples (9.6%), although sub-dominant at only 0.11% mean relative abundance. The type strain, Sanger_33^T^ ( = DSM 102154^T^), was isolated from human faeces. Its G + C content of genomic DNA is 48.7%.

## Supplementary Information

Supplementary Figures
