# Supplementary material for: Automated analysis of genomic sequences facilitates high-throughput and comprehensive description of bacteria
Source: ISME Commun. 2021 May 20;1:16. doi: 10.1038/s43705-021-00017-z (PMC9723785; doi:10.1038/s43705-021-00017-z)
Supplement: Supplementary file 1 — Supplementary Figures [file 43705_2021_17_MOESM1_ESM.docx]

**Supplementary Figures**

**
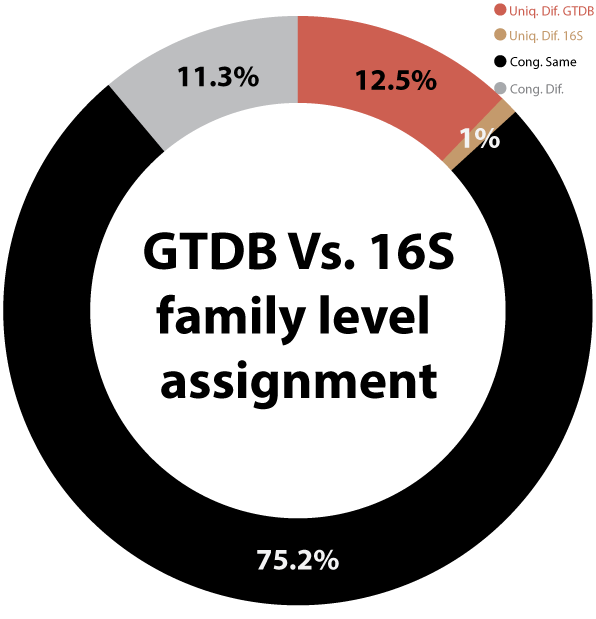
**

**Supplementary Figure 1. Family-level comparison of taxonomic delineation methods.** Pairwise comparisons to test the consistency of family-level delineation parameters (n = 30,247). Four groups were formed based on the consistency between GTDB and 16S rRNA gene sequence similarity assignment of the paired genomes: congruent results, same family (Cong. Same), congruent results, different families (Cong. Dif.) and those uniquely identified as belonging to different families according to the method specified (Uniq. Dif.). The uniquely different slices are coloured according to the method: GTDB (red), 16S rRNA gene similarity (brown), POCP (blue).
